# Depletion of Gram-Positive Bacteria Impacts Hepatic Biological Functions During the Light Phase

**DOI:** 10.3390/ijms20040812

**Published:** 2019-02-14

**Authors:** Hui Yun Penny Oh, Sandrine Ellero-Simatos, Ravikumar Manickam, Nguan Soon Tan, Hervé Guillou, Walter Wahli

**Affiliations:** 1 Interdisciplinary Graduate School, NTU Institute for Health Technologies, Nanyang Technological University Singapore, 50 Nanyang Avenue, Singapore 639798, Singapore; HOH001@e.ntu.edu.sg; 2 Lee Kong Chian School of Medicine, Nanyang Technological University Singapore, 11 Mandalay Road, Singapore 308232, Singapore; ravikumar_vet@hotmail.com (R.M.); NSTAN@ntu.edu.sg (N.S.T.); 3 INRA UMR1331, ToxAlim, 180 Chemin de Tournefeuille, 31300 Toulouse, France; sandrine.ellero-simatos@inra.fr (S.E.-S.); herve.guillou@inra.fr (H.G.); 4 School of Biological Sciences, Nanyang Technological University Singapore, 60 Nanyang Drive, Singapore 637551, Singapore; 5 Center for Integrative Genomics, University of Lausanne, Le Génopode, CH-1015 Lausanne, Switzerland

**Keywords:** circadian rhythm, liver, antibiotics, gut microbiota, gene expression

## Abstract

Living organisms display internal biological rhythms, which are an evolutionarily conserved adaptation to the environment that drives their rhythmic behavioral and physiological activities. The gut microbiota has been proposed, in association with diet, to regulate the intestinal peripheral clock. However, the effect of gut dysbiosis on liver remains elusive, despite that germfree mice show alterations in liver metabolic functions and the hepatic daily rhythm. We analyzed whether the disruption of gut microbial populations with various antibiotics would differentially impact liver functions in mice. Our results support the notion of an impact on the hepatic biological rhythm by gram-positive bacteria. In addition, we provide evidence for differential roles of gut microbiota spectra in xenobiotic metabolism that could protect against the harmful pharmacological effects of drugs. Our results underscore a possible link between liver cell proliferation and gram-positive bacteria.

## 1. Introduction

Holobiont is the ensemble of a eukaryotic host and its associated myriad of micro-organisms that live in symbiosis to adapt to their dynamic environment [1,2]. A typical human host harbors as much as 10 times more microbes than its cell number, and perplexingly, these microbes encode 100 times more unique genes than their host [3]. Over the past decade, a growing body of evidence that stresses the importance of this coexistence for the host’s health has emerged. In fact, the mammalian gut has an aggregate of trillions of non-pathogenic bacteria that interact mutually with their host [4], and that are affected by many cues, such as type and amount of food, feeding-fasting time, sleep and wake duration, exercise, and the light-dark cycle that entrains the host intrinsic biological rhythms in-sync with the external environment [5]. In a healthy host, the gut microbiota can support its survival by feeding on ingested nutrients and fermented products of otherwise indigestible dietary fibers that are found in the host colon [6]. Concomitantly, the host’s feeding behavior is a significant factor that regulates the diurnal oscillating composition and function of the gut microbiota [7,8].

In the homeostatic state, microbes help in the nutrient extraction from food, thereby producing various by-products that are useful for the host in oscillating concentrations, depending on the feeding rhythm. The absorbed by-products help to fine-tune the host biological functions. One example is the firmicute-derived short-chain fatty acids, which are ligands for many metabolic sensors that can regulate the expression of genes that are involved in metabolism [4,9,10]. In turn, the meal schedule impacts the daily clock in peripheral organs such as the liver, thus promoting the microbiota as a mediator between feeding and daily rhythm [11,12] and a player in metabolic- and chrono-related diseases [4,9,11]. Gut dysbiosis has been reported in shift workers and patients with sleeping disorders, whose body internal clocks are chronically desynchronized with the external light-dark cycle, predisposing them to diseases, such as obesity and/or diabetes [13]. Gut bacteria have multiple ways of interacting with their host, including communication with the brain in which several nuclear receptors are involved [14].

Studies have addressed the involvement of microbiota in peripheral organ daily rhythms. For instance, antibiotic administration results in a less diverse gut microbiota and it contributes to the perturbed circadian clock in small and large intestines that are directly exposed to the perturbed gut microbiota [15,16]. However, the effect of gut dysbiosis on the liver remains less known, although this organ receives 70% of its blood supply from the intestine through the portal vein [4], and it is therefore likely to be influenced by the microbiota composition and its products. Recently, using the germfree (GF) mouse model, we highlighted the perturbed expression pattern of the hepatic circadian core clock genes and clock effectors in these animals, especially during the light phase revealing the importance of the gut microbiota in maintaining the daily hepatic rhythm [17]. We hypothesized that specific spectra of the gut microbiota might regulate daily hepatic rhythm. In the present paper, we focus on the effects of selective depletion of different bacterial populations on the host’s liver physiology. We demonstrate that the hepatic clock is perturbed at Zeitgeber Time 4–6 (ZT4–6) after the vancomycin treatment of specific pathogen-free (SPF) mice. Similar perturbations were not observed in GF mice that were treated with vancomycin, suggesting that the eliminated bacteria, and not bacteria-independent pharmacological effects, are implicated in fine-tuning the hepatic clock and its effectors. We have also found that the different spectra of bacteria might be playing distinct roles in hepatic xenobiotic metabolism and some might have a protective effect on the liver. Lastly, our findings suggest that gram-positive bacteria impact pathways that are associated with cell proliferation and we speculate that these bacteria may impact liver regeneration and possibly tumor development.

## 2. Results

### 2.1. Faecal Microbiota Differs between Mice with Different Antibiotic Treatments

**Figure 1:**
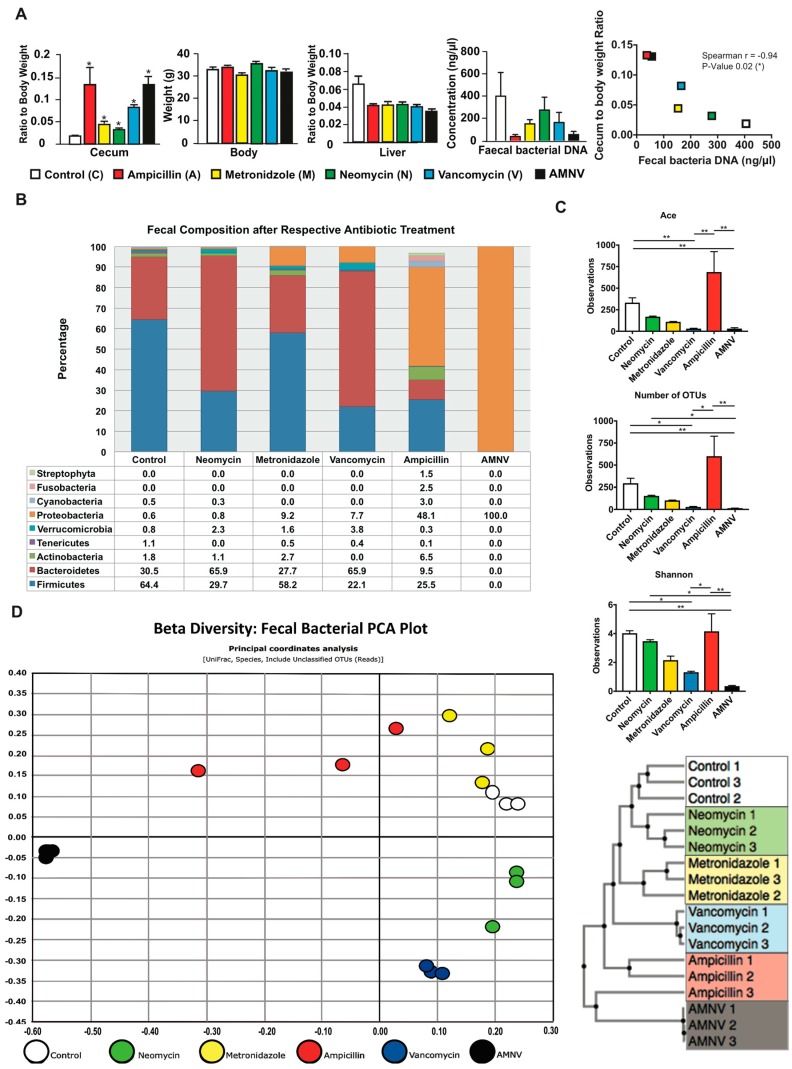
Phenotypic and fecal analysis of four-week antibiotic-treated specific pathogen-free (SPF) mice. (**A**) Average cecum to body weight ratio, body weight, liver to body weight ratio, bacterial DNA concentration in the extracts of faeces collected by direct defaecation, and faecal bacteria DNA load versus cecum to body weight ratio correlation plot are presented after the mentioned antibiotic treatments (*n* = 5, except for control cecum weight with *n* = 4, all faecal analysis with *n* = 3). Cecum and liver weight were normalized to respective average body weight before performing statistical analysis. Statistical analysis was performed using two-tailed Mann-Whitney test for all presented data except for the correlation between faecal bacteria DNA load and cecum to body weight ratio, which was calculated using two-tailed Spearman correlation. Data are presented as means ± standard error of the mean. Statistical significance from the respective controls is represented by * for *p*-value < 0.05. For Spearman correlation, negative r-value (−0.94) and *p*-value < 0.05 represent significant negative correlation. The color indication for each treatment group is represented at the lower panel. (**B**) Faecal samples were harvested before euthanization of the mice at ZT 4–6 and fecal microbiota was analyzed from 0.4–0.5 g of faeces after one month of antibiotic treatment. Relative abundance of faecal bacterial communities across different treatment groups with the color indication and percentage of each phylum defined on the bottom panel. Bacteria phyla with abundance of less than 1% are not shown. (**C**) Alpha diversity indicating species richness (abundance-based coverage estimator (ACE) and the number of operational taxonomic units (OTUs) found in Microbiome Taxonomic Profiling (MTP)) and diversity index (Shannon), as indicated. Kruskal–Wallis, followed by uncorrected Dunn’s multiple comparison were performed; statistically significant differences are indicated by * for *p*-value < 0.05 and ** for *p*-value < 0.01 (**D**) Beta diversity represented in the form of Faecal Bacteria Principal Component Analysis (PCA) plot and the Unweighted Pair Group Method with Arithmetic Mean (UPGMA) clustering tree. The different colors indicate different treatments as shown at the bottom of the PCA plot.

We have previously reported that GF mice display altered daily oscillation of clock gene expression and clock output regulators, with the most important perturbation being monitored during the light phase (ZT6) [17]. We hypothesized that the liver circadian clock would also be perturbed during the light phase around ZT6 in antibiotics treated mice. After one-month antibiotic treatment, all of the samples were collected during the light phase between ZT4 and ZT6. Interestingly, all the animals in the treated groups presented a significantly bigger cecum with an approximate two-fold or more increase in cecum weight (Figure 1A). A dilated cecum is an indicator of gut dysbiosis and it is also a characteristic phenotype of GF mice, which can be reversed upon the restoration of the gut microbiota [18,19]. There was no effect on body weight, but we observed a significant reduction in liver weight for all treatments after normalization to body weight (Figure 1A). We also noticed the enlargement of the gallbladder upon vancomycin and antibiotic combination (AMNV) treatments, as well as an abnormal fat distribution upon neomycin treatment (Figure S1).

Bacterial DNA was isolated from 0.4–0.5 g of faeces that were collected by direct defaecation at ZT4–6 before euthanization of the mice (Figure 1A). The highest amount of microbial DNA extracted was in the faecal samples of the untreated control group. When compared with this group, the DNA amount of the neomycin-treated group sample was moderately lower, while that of the vancomycin and metronidazole-treated groups was approximately 2.5-fold lower. The ampicillin and AMNV treatments caused a greater gut dysbiosis, with 13.9- and 7.3-fold reductions in the faecal DNA extracted, respectively, which correlated with higher relative ceca weight. In fact, the amount of bacterial DNA extracted from the faeces was significantly inversely correlated with the cecum to the body weight ratio (Figure 1A).

Next, we examined the faecal bacteria composition by 16S rRNA gene sequencing. The Good’s coverage index was higher than 97%, indicating that the global microbe diversity was mostly covered (Figure S2). Sequencing revealed significant differences in microbiota composition among the treatment groups (Figure 1B). The faecal composition in the control group was mainly Firmicutes (64.4%), Bacteroidetes (30.5%), and Actinobacteria (1.8%), which is in agreement with a previous report [20]. The neomycin-treated group microbiota was however dominated by Bacteroidetes (65.9%), followed by Firmicutes (29.7%), Actinobacteria (1.1%), and an additional phylum, Verrucomicrobia (2.3%). Firmicutes (58.2%), Bacteroidetes (27.7%), Actinobacteria (2.7%), Verrucomicrobia (1.6%), as well as an additional phylum, Proteobacteria (9.2%) dominated the microbiota isolated from metronidazole-treated mice. The vancomycin-treated group microbiota also presented major composition changes with Bacteroidetes (65.9%) and Firmicutes (22.1%) and two additional phyla Proteobacteria (7.7%) and Verrucomicrobia (3.8%). In this treatment group, Actinobacteria were not observed. The ampicillin-treated group microbiota that had the most drastic size reduction of the microbiota population was composed of Firmicutes (25.5%), Bacteroidetes (9.5%), and Actinobacteria (6.5%), and a high proportion of Proteobacteria (48.1%). Interestingly, many additional phyla of microorganisms, such as Streptophyta (1.5%), were observed in this treatment group, which is consistent with a previous report [21]. Prominently, many additional phyla were present at a minute amount (phyla < 1% are not shown in Figure 1B) after ampicillin treatment, resulting in the most varied bacteria composition in the faeces. Lastly, the faecal microbiota of the AMNV-treatment group was characterized by a dramatic expansion of Proteobacteria (nearly 100%), consisting of the Enterobacteriaceae *Escherichia coli*, suggesting the near to complete elimination of bacteria from other phyla.

The alpha diversity indices, which measure both the species richness (ACE, abundance-based coverage estimators; OTU, operational taxonomic unit) and diversity (Shannon index), indicated a reduction in both richness and diversity, particularly after the vancomycin and AMNV treatments. However, the microbiota of the ampicillin treatment group, which had the lowest fecal bacteria DNA load, retained a diversity similar to that of the control samples (Figure 1C), thus confirming a previous observation [21]. The beta diversity presented in the form of an unifrac principal coordinate analysis (PCoA) and a generalized unifrac Unweighted Pair Group Method with Arithmetic Mean (UPGMA) clustering tree is shown (Figure 1D). These results underscore the similarity in faecal bacteria composition in animals of the same antibiotic treatment group and the clear differences in the composition of the microbiota in mice from the different antibiotic treatment groups. Based on the beta diversity plot, the gut microbiota that were isolated after antibiotic treatment were different from the control microbiota, with AMNV being the most different, followed by ampicillin, vancomycin, metronidazole, and lastly neomycin being the closest to control. The AMNV treatment was particularly efficacious, resulting in the elimination of most of the faecal microbiota species, except for the multi-drug resistant Proteobacteria.

Collectively, these results demonstrate successful depletion of selective bacteria populations by different antibiotics. Vancomycin and AMNV treatment had a significant reduction in species richness and diversity (Figure 1C), with the AMNV group having the most different gut microbiota from that of the control group (Figure 1D).

### 2.2. Effect of Antibiotic Treatments on Hepatic Clock, Metabolism and Detoxification Gene Expression

**Figure 2:**
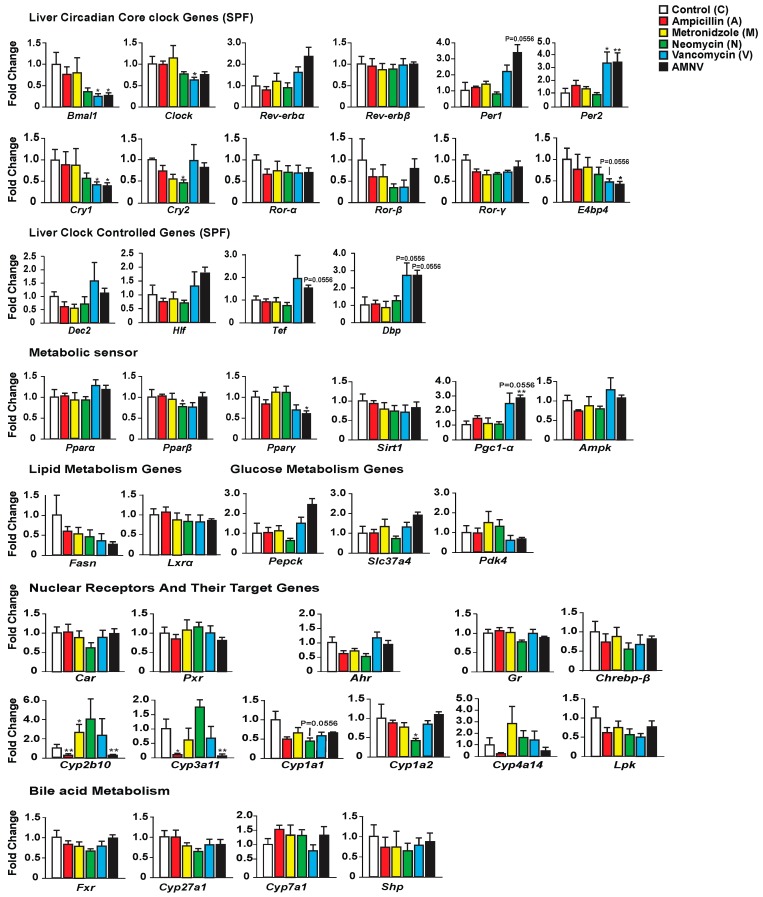
Hepatic gene expression analysis in SPF mice with the color indication for each treatment group on the top-right panel. Statistical analysis was done using the Mann–Whitney test (*n* = 5). Data are presented as means ± standard error of the mean. Significance relative to control is represented by * for *p*-value < 0.05, ** for *p*-value < 0.01.

We first investigated whether antibiotic-induced gut dysbiosis affects the expression of liver core clock genes and clock-controlled genes during the light phase at ZT4–6 (Figure 2). While ampicillin and metronidazole treatments had no effect when compared with control, the expression of some of these genes was modified in the three other treatment groups. The most substantial effect was seen after treatment with AMNV, followed by vancomycin and then neomycin. Neomycin treatment caused a significant reduction of *Cry2* expression. Vancomycin and AMNV treatments had a bigger impact and they were similar in causing the downregulation of *Bmal1*, *Clock* (a trend in AMNV), and *Cry1* expression, while *Per2* was upregulated significantly when compared to the control group. Several other clock or clock-associated genes showed similar upregulation trends, such as *Per1*, *Cry2*, and *Rev-erbα*, without reaching significance. We next tested whether the expression changes in the clock components also affected the expression of genes encoding clock output transcription factors, namely the basic helix-loop-helix repressor *Dec2* (*Bhlhb3*) and the PAR bZip factors *Dbp*, *Hlf*, and *Tef*. The expression of these four genes presented a similar upregulation trend in animals that were treated with vancomycin and AMNV (Figure 2). Together, these results show that antibiotics can perturb liver clock gene expression during the light phase and that vancomycin and AMNV treatments have relatively strong altering effects.

Next, we looked at the expression of several genes involved in energy metabolism that are known to display a daily expression pattern (Figure 2). The expression of *Pparβ*/*δ* and *Pparγ* was significantly lower after neomycin- and AMNV-treatment, respectively, while that of *Pparα* was not affected. Other key factors that mediate the crosstalk between the circadian clock and the metabolic pathways were also investigated (Figure 2). The expression of *Pgc-1α* was increased in both the vancomycin (trend; *p* = 0.0556) and AMNV-treated groups (*p* < 0.01), while *Sirt1* and *Ampk* expression were not changed by the antibiotic treatments. The limited impact of the treatments on liver energy metabolism genes at ZT4–6 is substantiated by the lack of a significant effect on the expression of two fatty acid metabolism genes (*Fasn*, *Lxrα*) and three glucose metabolism genes (*Pepck*, *Slc37a4*, and *Pdk4*). Notably, vancomycin and AMNV treatments induced similar trends, however without reaching statistical significance. We concluded that the antibiotics used had a relatively little impact on the expression of genes that are involved in energy metabolism during the light phase, although a global gene expression profiling may reveal genes that are affected more.

Finally, as antibiotics are xenobiotics, we explored their impact on genes participating in hepatic xenobiotic and bile acid detoxification. First, we assessed the expression of ligand-activated transcription factors (*Car*, *Pxr*, *Ahr*, and *Gr*), in addition to the *Ppars* mentioned above. The expression of none of these genes, including *Pparα*, was affected by the antibiotic treatments (Figure 2). It was of interest to test whether the expression of selected target genes of these receptors was affected by the treatments, since the activity of these receptors is ligand-dependent, and the ligands may be impacted by the treatments. One important class of genes these receptors regulate is coding for phase I xenobiotic enzymes, such as the cytochrome P450s (CYPs) [22]. Interestingly, we indeed found that some target genes of these receptors (in brackets below), for instance, *Cyp2b10* (CAR), *Cyp3a11* (PXR), and *Cyp4a14* (PPARα & GR), showed a similar downregulation in the ampicillin- and AMNV-treated mice. Thus, the effects that were observed upon AMNV treatment may be caused by ampicillin. On the contrary, neomycin caused an upregulation trend of *Cyp2b10* and *Cyp3a11*. Interestingly, both the well-absorbed metronidazole and the poorly absorbed vancomycin did not affect xenobiotic metabolism. Neomycin, which is another poorly absorbed antibiotic, downregulated the AHR target genes, *Cyp1a2* and *Cyp1a1*. Collectively, these results show that antibiotics have a significant and diverse impact on liver xenobiotic metabolism genes, which does not depend on the antibiotic absorption characteristics.

### 2.3. Antibiotic-Associated Daily Rhythm Disruption during Light Phase in SPF Mice is Mediated by the Gut Microbiota

The impact on liver gene expression, as measured at ZT4–6 in vancomycin and AMNV treated mice, could be due to (1) an antibiotic-induced depletion of crucial bacteria that regulate host liver daily rhythm, (2) antibiotic non-susceptible bacteria that remained in the gut or increased presence of antibiotic-resistant bacteria, and (3) a bacteria-independent pharmacological effect of the antibiotics.

**Figure 3:**
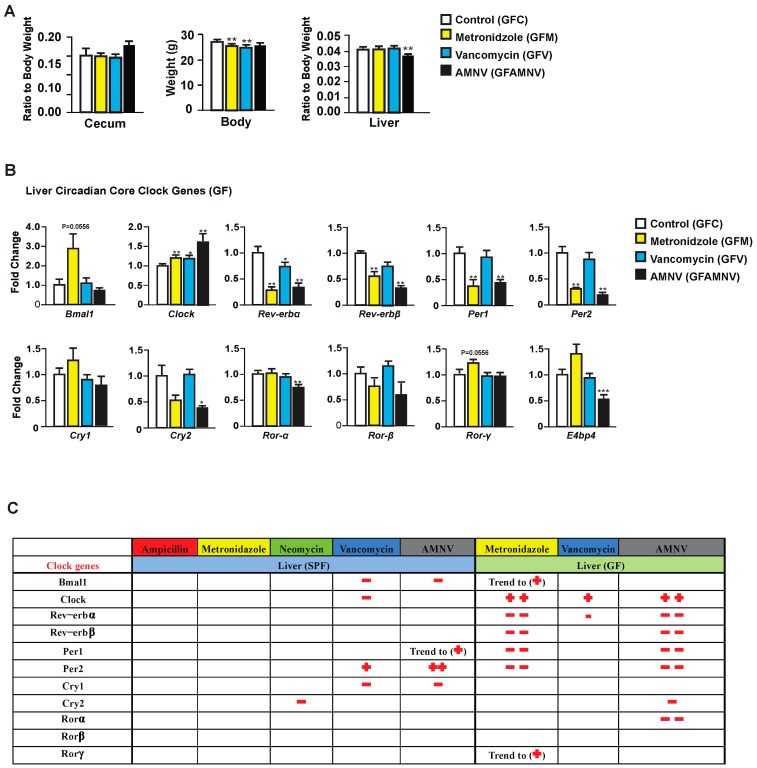
Phenotype and liver core clock gene expression of antibiotic-treated germfree (GF) mice. (**A**) Average cecum to body weight ratio, body weight and liver to body weight ratio of GF animals after four weeks of the respective antibiotic treatments (*n* = 10, except for cecum measurement in the GF vancomycin and antibiotic combination (GF AMNV) treatment group with *n* = 9). (**B**) Hepatic gene expression analysis in GF mice. Expression of selected genes with the color indication for each treatment group (see the color code on the right of the panel). Statistical analysis was done using the Mann-Whitney test (*n* = 5). Data are presented as means ± standard error of the mean. Significance relative to control is represented by * for *p*-value < 0.05, ** for *p*-value < 0.01, *** for *p*-value < 0.001. (**C**) A summary comparing changes in liver clock gene expression after antibiotic treatment in SPF and GF mice (see Figure 5). Downregulation is represented by the − sign in red while upregulation is represented by + in red and the number of symbol indicates the level of statistical significance, with one symbol indicating a *p*-value < 0.05, two symbols indicating a *p*-value < 0.01, and three symbols a *p*-value < 0.001. Close to statistical significance data are shown as Trend to (−) or (+).

To explore the possible pharmacological effects of metronidazole, vancomycin, and AMNV on gene expression, we treated GF mice—reported to have a liver daily rhythm different from the SPF mice due to the lack of regulation coming from the gut microbiota [17]—with these antibiotics and compared the treated GF mice with their untreated counterparts. As for the SPF mice, we first analyzed the phenotype of the GF treated mice (Figure 3A). Upon one month of antibiotic treatment, we observed a significant reduction in body weight of GF mice after metronidazole and vancomycin treatment, which was not observed in the SPF mice. The relative weight of the cecum, as expected, did not significantly differ after antibiotic treatment in the GF mice, but the ceca in these mice were at least five times heavier than in SPF mice, due to the absence of microbiota. Unlike the SPF mice, one month of metronidazole or vancomycin treatment did not have any significant effect on the GF liver weight. However, we saw a reduction in liver weight in GF mice after AMNV treatment, but this reduction was much smaller than in the SPF mice. It is noteworthy that the liver weight to body weight ratio was higher in non-treated SPF (0.06) compared to non-treated GF mice (0.04), while after antibiotic treatment of SPF mice, this ratio was similar to that found in the untreated and treated GF mice (0.04). Collectively, these results suggest a novel role of the microbiota in regulating the relative liver weight and that its reduction observed after metronidazole and vancomycin treatment is due to the elimination of parts of the microbiota and not to a pharmacological effect of the antibiotics, because the liver weight of antibiotic-treated GF mice was not reduced.

Next, we looked into the expression of liver core clock genes in GF mice after antibiotic treatment (Figure 3B) and compared it to that of SPF mice (Figure 2). Unlike in the SPF mice, the expression of *Bmal1*, *Per2*, *E4bp4*, and *Cry1* were unaffected and that of *Clock* was affected in an opposite manner in GF mice. We also observed that *Rev-erbα* was significantly downregulated in the GF mice after vancomycin treatment, which was not observed above in the SPF mice. These observations demonstrated that, in SPF mice, it is the microbiota modification caused by vancomycin that is the causal factor for the changes in gene expression in the clock machinery. However, as shown in GF mice, vancomycin also had a pharmacological effect that is different from that caused by altered gut microbiota.

Peculiarly, metronidazole had a strong pharmacological effect on liver clock gene expression in GF mice, an effect that was not observed in the SPF mice. Out of 11 core clock genes investigated, seven were affected, five significantly and two with a p-value of 0.055. The gene expression in GF mice after AMNV treatment resembled that of metronidazole treated GF mice (Figure 3B), suggesting a dominant effect of metronidazole in the combination treatment.

From these results, we concluded that the perturbation of the hepatic gene expression that was observed during the light phase in the SPF mice after vancomycin treatment was due to gut microbiota dysbiosis. On the contrary, metronidazole had a strong pharmacological effect in GF mice, whereas the SPF mice with their microbiota seemed to be protected from this pharmacological effect.

### 2.4. Antibiotics-Induced Differential Changes in the Post-Transcriptional Regulation of Circadian and Metabolic Genes

**Figure 4:**
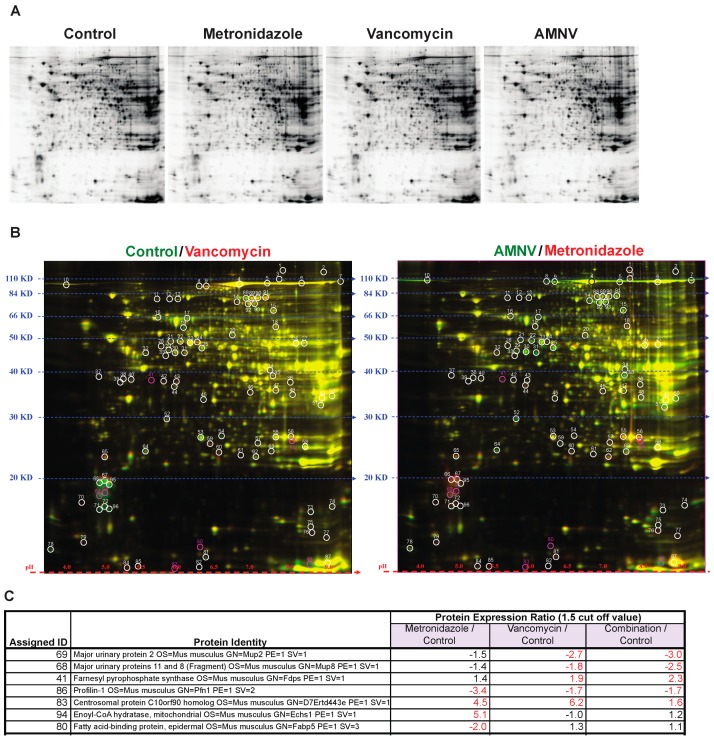
Gel electrophoresis analysis of protein lysates from livers of mice in the different antibiotic treatment groups. (**A**) Proteins were separated by sodium dodecyl sulfate polyacrylamide gel electrophoresis (SDS-PAGE) according to molecular weight and isoelectric charge. (**B**) On the right, two-dimensional fluorescence difference gel electrophoresis (2D-DIGE) image of control lysate proteins labeled with Cy-3 (green) and vancomycin lysate protein labeled with Cy-5 (red), and on the left, image of AMNV lysate proteins labeled with Cy-3 (green) and metronidazole lysate proteins labeled with Cy-5 (red). Molecular weight and isoelectric points are indicated on the left and bottom of the image, respectively. Spots in red or green represent proteins with increased expression in vancomycin and metronidazole, and control and AMNV lysates, respectively, while the yellow spots represent proteins with similar expression in the livers of control and vancomycin or AMNV and metroniazole treated mice. Differentially expressed spots are circled and assigned a number for the ease of identification. Fold change of protein expression was determined by DeCyder analysis and the selected spots highlighted in pink were identified by mass spectrometry. (**C**) List of assigned protein IDs identified by mass spectrometry and their relative expression ratio (treated vs control). A larger than 1.5 times change in expression was considered to be significant (indicated by fold change in red). See Supplementary Table S2 for the list of all protein spots.

The impact of antibiotics on gene expression at the mRNA level reported above prompted us to analyze liver proteins after antibiotic treatments, as antibiotics may also affect post-transcriptional regulations. To address this question, we selected three treatment groups for analysis based on the liver mRNA expression profiles in SPF mice, specifically for mice that were treated with metronidazole that did not affect liver circadian genes, and with vancomycin and AMNV that both significantly affected liver circadian genes (Figure 2). We limited the study to SPF mice, which are more relevant for this study. Using Two-Dimensional Fluorescence Difference Gel Electrophoresis (2-D DIGE), we compared the protein expression ratio between the antibiotic-treated with the non-treated control groups (Figure 4A,B, Table S2). Spots were selected based on protein expression ratio with the cut-off set at 1.5 (Table S2). Mass spectrometry was used to identify the proteins in the selected spots (Figure 4C).

We found that the major urinary proteins (MUPs) 2, 8, and 11, which belong to the lipocalin superfamily, were downregulated significantly in only the vancomycin and AMNV treatment groups. Another protein that, on the contrary, was only upregulated in the vancomycin and AMNV groups is farnesyl pyrophosphate synthase (FPPS), an enzyme that synthesizes farnesyl pyrophosphate, a key intermediate in the daily regulated cholesterol and sterol biosynthesis [23,24].

Aside from the proteins mentioned above that were regulated by both the vancomycin and AMNV treatments, we observed an upregulation of centrosomal protein C10orf90 homolog (FATS) (1.6–6.2×) and downregulation of profilin-1 (Pfn1) (1.7–3.4×) in all three treatments tested. Interestingly, the genes coding all the abovementioned proteins were not regulated at the mRNA level (not shown). This observation demonstrated post-transcriptional effects of the antibiotics.

Although treatment with metronidazole did not affect the mRNA level on the expression of genes that are associated with energy metabolism, we found an upregulation (5.1×) at the protein level of enoyl-CoA hydratase (ECHS) and a downregulation (2×) of fatty acid binding protein 5 (FABP5). ECHS is essential in metabolizing fatty acids in β-oxidation to produce both acetyl CoA and energy in the form of ATP. FABP5 is important for the intracellular uptake of fatty acids (Figure 4C).

In brief, this proteomics analysis revealed additional liver functions on which the microbiota has an impact via post-transcriptional regulation. We found that the microbiota downregulated proteins that were implicated in liver signaling to other organs and sexual/aggressive behavior (MUPs; vancomycin, and AMNV), cell cycle progression (PFN1; metronidazole, vancomycin, and AMNV) and fatty acid uptake (FABP5; metronidazole), and upregulated proteins of cholesterol and sterol biosynthesis (FPPS; vancomycin and AMNV), cell cycle progression (FATS; metronidazole, vancomycin and AMNV), and fatty acid metabolism (ECHS; metronidazole). Therefore, antibiotics exert a significant posttranscriptional effect.

### 2.5. Antibiotics Cause Changes in Liver Metabolite Profile

**Figure 5:**
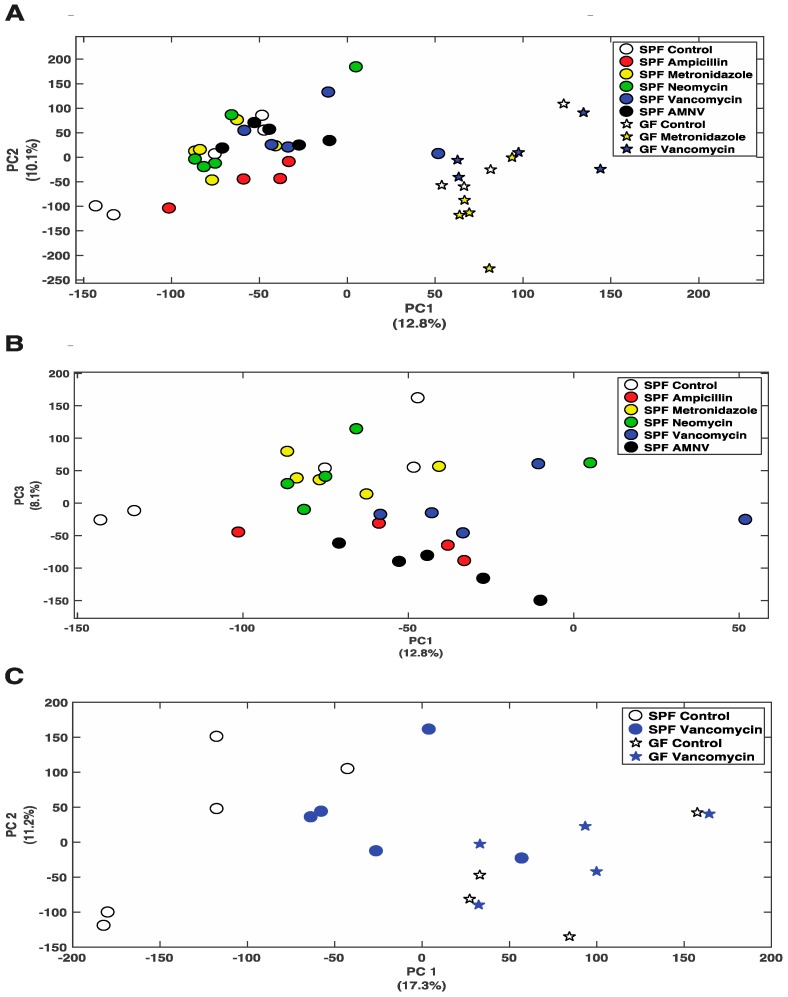
Nuclear Magnetic Resonance (NMR) analysis of polar metabolites isolated from liver extracts. (**A**) PCA plot showing the distribution of SPF and GF mice, with colored symbols corresponding to the different treatments given on the right of the panel. (**B**) PCA plot showing the distribution of SPF mice with colored symbols corresponding to the different treatments given on the right of the panel. (**C**) PCA plot showing the distribution of SPF and GF mice that were treated with vancomycin, as well as their untreated counterparts, with colored symbols for the different treatments given on the right of the panel. For all PCA plots, the percentage of variance explained by each axis is indicated next to its axis title.

So far, we have shown that antibiotics can affect gene expression at the mRNA and protein levels. Next, we asked whether the differential removal of gut microbiota or its complete absence in GF mice would change the liver metabolite profile. Using Nuclear Magnetic Resonance (NMR) spectroscopy, we analyzed the effects of all five treatments in SPF mice and the metronidazole, and vancomycin treatments of GF mice. Principal Component Analysis (PCA) that was obtained from the various groups, including the control groups, showed a distinct separation between SPF and GF mouse liver metabolites (Figure 5A). Consistent with the fecal data, ampicillin, vancomycin, and AMNV treated SPF mice had their liver metabolite profiles separated from the control mice (Figure 5B). This separation was particularly evident for the vancomycin treatment in SPF mice, for which we saw the strongest effect so far at the mRNA and protein levels, with an important shift towards the metabolite profile in GF mice (Figure 5C).

**Figure 6:**
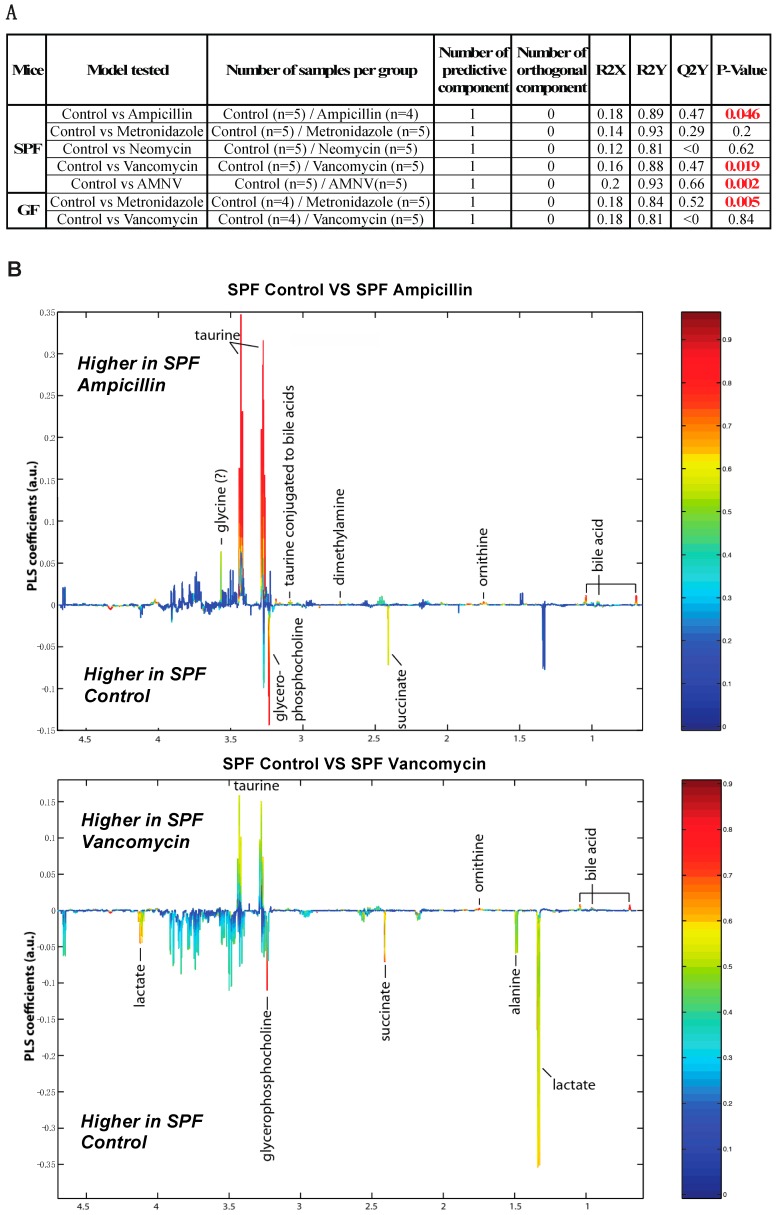

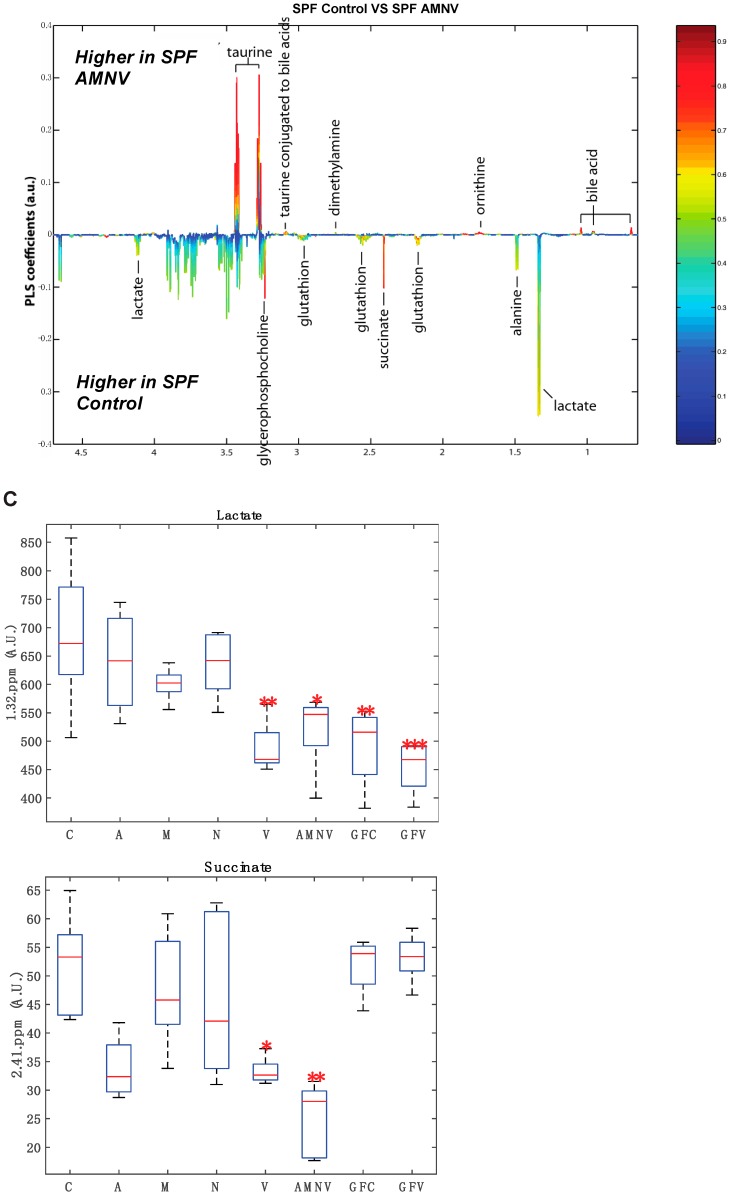

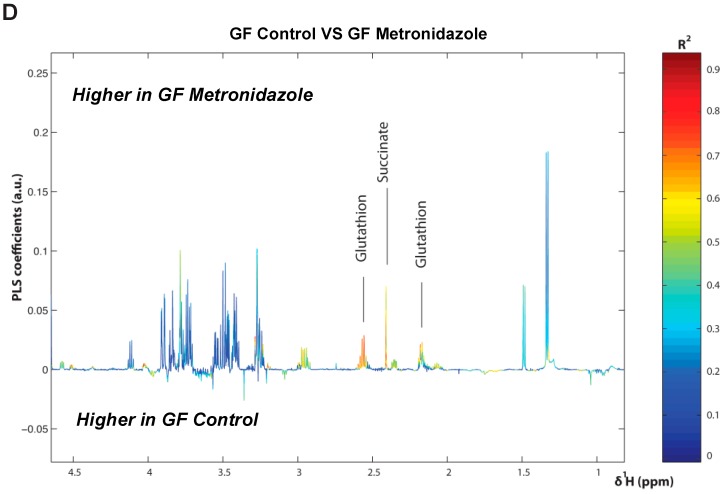
Liver metabolite profiles of SPF and GF antibiotic treatment groups. (**A**) A summary table showing all of the parameters used for the generation of orthogonal projection on latent structure-discriminant analysis (O-PLS-DA) plots and quality of the results of O-PLS-DA generated for all the SPF and GF models. The number of samples used for the respective groups is indicated in brackets together with the number of predictive and orthogonal components used. R2X, R2Y, and Q2Y values for each O-PLS-DA generated are shown. Significant *p*-values that were generated using student *t*-test are represented in red. (**B**) O-PLS-DA correlation coefficients (r2) maps that tested to be significant by student *t*-test are presented. R2 map discrimination between polar liver metabolite extracted from SPF Control (C) vs. SPF Ampicillin (A), SPF Control (C) vs. SPF Vancomycin (V) treated group, and SPF Control (C) vs. SPF AMNV treated group. Peaks in the positive upward direction indicate metabolites that were more abundant in the treated groups, while metabolites that were more abundant in control groups are presented as peaks in the negative downward direction. (**C**) Boxplots of lactate and succinate for antibiotic-treated groups, as indicated. One-way ANOVA analysis was performed on NMR lactate’s signature peak at position 1.32 ppm, as well as succinate with its signature peak at 2.41 ppm. Groups with statistically significant changes as compared to the SPF control are represented by * for *p*-value < 0.05, ** for *p*-value < 0.01, and *** for *p*-value < 0.001. Two outliers, one from SPF ampicillin treatment and one from GF non-treated control, were removed before the analysis. SPF control and respective antibiotic treated groups are represented by C (Control), A (Ampicillin), M (Metronidazole), N (Neomycin), V (Vancomycin) and AMNV (combination of all four antibiotics) while GF control and Vamcomycin-treated mice are represented by GFC and GFV respectively. (**D**) O-PLS-DA correlation coefficients (r2) map tested significant by student *t*-test is presented. R2 map discrimination between polar liver metabolite extracted from GF control vs. GF Metronidazole. One outlier sample from GF control treatment was removed. Peaks in the positive upward direction indicate metabolites that were more abundant in treated groups, while metabolites that were more abundant in control groups are presented as peaks in the negative downward direction.

To further this liver metabolite analysis, we fitted orthogonal projection on latent structure-discriminant analysis (O-PLS-DA) models of treated SPF and GF liver metabolites as compared to their respective untreated counterparts. Statistical parameters for each model are reported in Figure 6A. Interestingly, we observed a significant effect of AMNV, vancomycin, and ampicillin treatment on liver metabolites in SPF mice, while metronidazole treatment only significantly affected GF mice’s liver metabolites. We investigated the metabolites that are responsible for these discriminations using the O-PLS-DA loading plots (Figure 6B,D). The most significantly affected metabolites that were identified in the vancomycin-treated liver were also changed in the ampicillin and AMNV treatment groups, in which gram-positive bacteria were partially or entirely eliminated, respectively. We narrowed our analysis by focusing on vancomycin and AMNV treatments, both of which had the strongest effect on the daily parameters studied so far (refer to Table S3 for the list and details of the metabolites observed in the NMR spectra). We identified two metabolites whose downregulation was confined to the vancomycin and AMNV treatments: lactate and succinate (Figure 6B). Interestingly, in the GF mice, lactate was also significantly lower in the livers of the control mice and remained at the same low level after vancomycin treatment, similar to the lactate level that was observed in vancomycin-treated SPF mice (Figure 6C). This result suggests that the reduction of lactate was due to dysbiosis and not to the potential pharmacological effect of the antibiotic. Succinate level in GF mice, before or after treatment, closely resembled that of the liver of SPF untreated mice, thus also eliminating the possible pharmacological effect of vancomycin. These results suggest that the reduction of succinate in the vancomycin and AMNV treated SPF groups was due to a microbiota alteration, possibly the elimination of gram-positive bacteria. In line with this proposition, we also observed a near significant reduction in succinate after ampicillin treatment, a broad-spectrum antibiotic that eliminates gram-positive as well as gram-negative bacteria (Figure 6C).

In summary, based on our GF and SPF liver metabolite profiles, we conclude that gut microbiota has an important impact on the liver metabolite profile. In addition, we observed significantly lower levels of lactate and succinate upon the depletion of gram-positive bacteria, and this is unlikely caused by the pharmacological effect of the antibiotics used.

## 3. Discussion

The importance of the microbiota playing a role in influencing the gut daily rhythm and maximizing nutrient uptake by the host is known [25,26]. Antibiotics have been used extensively in various studies aiming at assessing the impact of gut microbe alterations in chronic inflammatory diseases, diabetes, and metabolic syndrome [27,28]. Furthermore, previous comparisons of SPF with GF mice have established that gut microbiota augments the absorption of energy from food and impacts daily liver oscillation [17,29,30]. It is noteworthy that GF mice have an altered daily rhythm of gene expression that is particularly pronounced at ZT6, possibly due to energy insufficiency during the resting phase [17].

The current work demonstrates the beneficial role of a gram-positive rich microbiota in SPF mice during the light phase. It shows that gut microbiota depletion by antibiotics affects several important hepatic functions, such as core clock gene expression, xenobiotic gene expression, lipid, and fatty acid metabolism, cell cycle progression regulatory pathways, and metabolite distribution. We observed a microbiota impact at the different functional levels of gene expression: mRNA, protein, and eventually metabolite distribution.

### 3.1. Selective Reduction in Microbiota by Antibiotics

The significant enlargement of ceca demonstrated the success of the antibiotic treatments in causing dysbiosis. Different antibiotic treatments successfully induced a differential elimination of gut microbes, as shown by the reduction in the faecal bacterial DNA concentration and changes in faecal microbiota composition, which were altered differently among the treated groups. We also observed a general reduction in bacterial diversity for all groups of antibiotic-treated SPF mice (significant for the vancomycin- and AMNV-treated groups), except for the ampicillin-treated mice, for which the bacterial alpha diversity index was similar to their untreated counterparts. Beta diversity plots indicated the similarity of faecal composition in mice that were treated with the same antibiotic and clear differences between mice in the different treatment groups. Furthermore, faecal microbiota composition from the control mice and mice that were treated with metronidazole, neomycin, and vancomycin were similar to those found in a previous study [31]. Ampicillin, a broad spectrum antibiotic, caused the greatest reduction in the amount of microbial DNA extracted from faeces, as consistent with a previous finding that ampicillin greatly reduces gut bacterial density [32]. Despite a drastic reduction in bacterial concentration, 16s rRNA gene sequencing and alpha diversity indices revealed a great number of different phyla in the ampicillin-treated faecal samples. A possible explanation for this observation could be the proliferation of opportunistic bacteria that thrive in a situation of profound gut dysbiosis. Finally, the faecal microbiota from the AMNV-treatment group was characterized by a dramatic expansion of Proteobacteria (nearly 100%). Blooming of Proteobacteria is a recognized signature of gut dysbiosis [33], which is increased after our antibiotic treatments. Hence, our fecal analysis supported a clear differential elimination of bacteria by different antibiotics.

### 3.2. Vancomycin Treatment-Induced Liver Clock Perturbation at ZT4–6 is due to Dysbiosis

Strikingly, vancomycin—a narrow spectrum glycopeptide antibiotic that eliminates gram-positive bacteria—perturbed the liver clock at the ZT4–6 time point that was chosen for this study based on our previous work [17]. Several clock genes (*Bmal1*, *Clock*, *Per2* and *Cry1*), proteins (FPPS, MUP2, 8 and 11) and hepatic metabolites (lactate, succinate) were significantly affected (mostly downregulated) upon vancomycin treatment (Figure 2, Figure 4 and Figure 6). The gut microbiome is known to produce clock modifying metabolites that drive liver transcriptional reprogramming and metabolite rhythms [34]. Our findings are consistent with our previous publication, which demonstrated the regulation of liver daily rhythm by the gut microbiota in a study using GF mice [17]. Importantly, it extends these results to additional liver functions. The gut microbiota is known to be involved in the circadian homeostasis of cholesterol by impacting the expression of genes that are involved in its synthesis and excretion [35]. Our results on the upregulation of FPPS that was observed in both the vancomycin and AMNV groups, together with supporting evidence from a previously published paper showing higher FPPS expression in GF mice as compared with SPF mice [35], suggest that vancomycin has eliminated a subpopulation of microbiota that participates in the circadian regulation of cholesterol metabolism. Of note, the AMNV-treatment group, characterized by a dramatic expansion of Proteobacteria (nearly 100%), also has an affected liver daily rhythm. However, based on the sequencing data of the ampicillin and vancomycin treatment groups, we ruled out Proteobacteria as the casual factor for liver daily rhythm disruption. In fact, Proteobacteria expansion (51.7% of the gut microbiota) was observed after ampicillin treatment that did not affect the daily rhythm, while vancomycin did it in mice having only a mere 7.6% of Proteobacteria. Since vancomycin is poorly absorbed, the effects that were observed are likely the consequence of vancomycin-induced dysbiosis. Despite a drastic reduction of the bacteria DNA load being observed after ampicillin treatment, the liver circadian rhythm was intact. This observation indicates that bacterial load is not too critical, but it is the type of bacterial species that matters. The impact on the liver would most likely be directly mediated by the enterohepatic circulation or indirectly via the systemic circulation. It is also worth mentioning that Actinobacteria, a gram-positive phylum, is depleted in both the vancomycin and AMNV groups, identifying Actinobacteria as a potential contributor in the regulation of liver daily rhythm, but this will have to be demonstrated in future work. The final proof and detailed mechanism by which gram-positive bacteria regulate the liver daily rhythm and liver functions should come from further studies, including the transplant of gram-positive bacteria in animals that are devoid of gut microbiota. We speculate that this regulation by gram-positive bacteria could be mediated in part by factors influencing hepatic *Pgc-1α* expression, as this effect was only seen in vancomycin-treated mice. Supporting our speculation, the *Pgc-1α* mRNA transcript level in liver primary hepatocytes is inversely proportional to that of *Bmal1* mRNA [36]. In addition, the enlarged gallbladder in the SPF vancomycin treated group indicates that bile acid circulation may participate in the crosstalk between microbiota and liver. Furthermore, the gut dysbiosis-induced reduction in the hepatic level of MUPs, lactate, and succinate can have systemic effects [37,38,39]. Daily regulated MUPs, which belong to the lipocalin superfamily, are mainly synthesized by the liver [40]. Upon their release into the bloodstream, MUPs bind to and stabilize pheromones that regulate the aggressive and sexual behaviors of mice via their excretion into the urine [41]. In addition, they are mediators involved in organ crosstalk, signaling via the bloodstream, between the liver and other organs [42]. A recent study has revealed that Mup1, which is produced by the hepatocytes, is lower in the serum of GF mice, supporting our observation that microbiota could be playing a role in its production [37]. Besides, daily activities and nutritional and health status are known to influence lactate, a potent signaling molecule that could potentially impact several organs, such as the brain, heart, and liver [43]. Lactate in the liver was reduced upon vancomycin treatment in the SPF mice, and the levels of lactate were similar to those found in the livers of GF mice before and after vancomycin treatment. This observation suggests that bacteria eliminated by vancomycin rather than pharmacological effects are involved in the control of the hepatic lactate levels. However, we cannot exclude the possibility of an indirect dysbiosis effect via extrahepatic organs. One might also argue that some of the effects of antibiotics treatment might come from an important change in the eating pattern of the treated mice. If this were the case, then we would expect similar changes in germfree mice as well. The fact that we did not, together with no significant change in weight in SPF mice after antibiotic treatment, suggest that it is most likely not a change in feeding pattern that causes the observed modifications at the mRNA, protein, and metabolite profiles.

### 3.3. Microbiota Protects against Metronidazole-Induced Circadian Perturbation

The oscillating composition of gut microbiota is known to influence the potency of xenobiotics whose metabolism is under the influence of the liver daily rhythm [36,44]. Xenobiotic sensors are intertwined in a complex network in which they may regulate one another [45]. For instance, the ligand-activated glucocorticoid receptor (GR) regulates the expression of two master xenobiotic sensors, namely, CAR and PXR, as well as other sensors, such as PPARα [45,46,47]. Herein, we show that different antibiotic treatments have distinct effects on the xenobiotic gene expression profiles at ZT4–6. Furthermore, the selective effects of these treatments lead to a marked difference in gut microbiota diversity. Out of all the antibiotics tested, it appears that ampicillin has the most impact on xenobiotic metabolism, with *Cyp2b10* and *Cyp3a11* being significantly downregulated, which is similar to previous observations that were made in GF mice [48]. On the contrary, treatment with metronidazole upregulated *Cyp2b10* and treatment with neomycin downregulated *Cyp1a2*. It is noteworthy that, based on the comparison made with GF mice, we found that the gut microbiota in SPF mice has a protective role against liver daily rhythm disruption by metronidazole. It is of interest that antibiotics could have an indirect impact on xenobiotic action through the disruption of the gut microbiota. This result highlights the potential of microbiota transplantation as a novel intervention against adverse drug responses in certain groups of people [49]. Our findings further underscore the importance of reassessing drug prescription to patients with gut dysbiosis.

### 3.4. Visceral Adiposity Observed upon Neomycin Treatment

In relation to obesity, Firmicutes and Bacteroidetes, which are the two main phyla of bacteria dominating the gut, have always been in the center of debate as to which population is the pivotal group in contributing to obesity in mammals. Obese mice and humans have been reported to have a higher ratio of Firmicutes to Bacteroidetes, although there are many controversial findings challenging this concept [4,50,51]. Independent of body mass index, individuals with central obesity have been reported to have a higher risk of metabolic and cardiovascular diseases [52]. Incidentally, Firmicutes were drastically reduced to less than 30% in neomycin, vancomycin, ampicillin, and AMNV groups, while Bacteroidetes bloomed to more than 65% in two of them, namely in the neomycin- and vancomycin-treated groups. Unlike what we observed after vancomycin treatment, a lower Firmicutes to Bacteroidetes ratio after the neomycin treatments did not correlate to a lean phenotype, as we noted an increase in the deposition of visceral fats restricted only to this treatment group. The buildup of visceral fats in this group, without a significant weight gain, came along with the significant perturbation of liver circadian and lipid metabolism genes, such as *Cry2* and *Pparβ*/*δ*. Taking the analysis to the family level (Table S4), gram-positive Lachnospiraceae (22.74%) dominated the Firmicute phylum, while S24-7_f (50.91%) dominated the Bacteroidetes phyla. Supporting our finding, the Lachnospiraceae family of bacteria has been associated with central obesity in twin studies [52], while the S24-7 family increased three-fold upon high-fat diet feeding in the mouse [53]. S24-7_f flourishing was not seen in other antibiotic treatment groups, indicating that its presence could be contributing to the buildup of visceral fats that are induced by neomycin, but this remains to be directly demonstrated. Based on these finding and previous contradictory findings, we suggest that visceral fats can be associated with microbes that belong to either Firmicutes or Bacteroidetes. A deeper analysis down to at least to the family level would be essential for unveiling the key players that contribute to visceral fat accumulation.

### 3.5. Regulation of Liver Regeneration by the Gut Microbiota

A question that is raised by our findings is whether microbiota could promote hepatic regeneration. We observed a downregulation of a protein that is involved in cell cycle progression (PFN1; metronidazole, vancomycin, and AMNV), as well as the upregulation of another protein that is also involved in cell cycle progression (FATS; metronidazole, vancomycin and AMNV). PFN1, which was downregulated, is a ubiquitous cytosolic actin-sequestering protein that is known for its role in the cytoskeleton dynamic and cell signaling [54]. To date, a potential role of PFN1 in early liver cancer progression has not been reported. However, the upregulation of PFN1 is known to be positively associated with cell cycle progression by promoting liver regeneration upon hepatectomy [55]. Here, its downregulation may suggest an inhibitory effect of antibiotics on cell cycle progression. FATS, which was upregulated, is an E2-independent ubiquitin ligase that promotes the p53-dependent transcription of p21 (a negative regulator of the cell cycle), leading to cell cycle arrest after DNA damage [56]. It was also previously shown that elevated FATS further promotes the acetylation of p21, which protects p21 from proteasomal degradation, modification by oncogenic kinases, as well as the maintenance of p21 stability in the nucleus [57]. Stabilization of p21 in the nucleus would lead to an impediment in cell cycle progression. In fact, the downregulation and upregulation of PFN1 and FATS, respectively, suggest that metronidazole, vancomycin, and AMNV may retard hepatic cell cycle progression.

The role of microbiota in liver regeneration has been documented though not well explored. Gram-positive *Lactobacillus* and *Bifidobacterium*, as well as gram-negative *Bacteroides* species, have been proven to aid in liver regeneration [58]. In addition, diurnal changes in liver mass and hepatocyte size has been linked to feeding cycles [59]. Interestingly, the ratio of liver to body weight that is presented in this recent study is very similar to our observation, with a ratio of 0.06 for livers that were harvested at ZT0 (similar to SPF mice control harvested at ZT4) and 0.04 for livers that were harvested at ZT12 (similar to antibiotic-treated SPF mice and GF with or without antibiotic treatment). To the best of our knowledge, the current study is the first to show that antibiotic treatments have an impact on liver mass, which is likely through perturbing commensal gut bacteria, which would impact normal liver turnover. This is corroborated by our antibiotic-treated GF mice, which did not demonstrate any reduction in weight or liver to body weight ratio when compared to their untreated counterparts, indicating a cause other than a pharmacological effect of the drugs. This finding might serve as a platform for more research into targeting gram-positive bacteria for promoting liver regeneration as well as prompting more research into understanding how bacteria can regulate liver mass. Our results also create awareness of potential liver hypotrophy that is associated with the use of antibiotics to treat bacterial infection in patients.

In summary, different compositions of gut microbiota differently impact liver physiology. Metronidazole in SPF mice causes skeletal muscle atrophy and it modifies the expression of genes that are involved in metabolic regulation and muscle clock machinery tuning [60]. Herein, we have shown that gram-positive bacteria play a role in regulating rhythmic liver functions. We speculate that bacteria may impede liver regeneration and may even have an anti-tumorigenic effect. This hypothesis has to be tested in future experiments. More work has to be done to identify the strains of bacteria that are responsible for the observed effects [61,62]. Our results also suggest that the specific alteration of gut microbiota might impact liver size, fat distribution, and deposition. Last but not least, our antibiotic treatment study casts some light on the possibility of developing less invasive methods, such as probiotics or microbiota transplants in the prevention or even treatment of daily rhythm related disorders. One limitation of the present study is that we have conducted the experiment at a defined period of the day. A much bigger and more comprehensive similar study for the future would be to do a time series at a resolution of four-hour intervals to gain more insight into the effects of the antibiotics over the whole 24-hour daily cycle. Such a study would also allow for addressing the possible impact of antibiotics on the structure and function of the endogenous mammalian clock machinery, including its circadian feedback loop and its putative physiological consequences [63].

## 4. Materials and Methods

### 4.1. Animal Care and Experimental Procedures

All of the animal experimentation protocols were approved by the Institutional Animal Care and Use Committee (IACUC), Singhealth, Singapore (2015/SHS/1133; 24th December 2015).

### 4.2. Animals

C57BL/6J mice acquired from Jackson Laboratory (Bar Harbor, ME, USA) were maintained at the Singhealth Experimental Medicine Centre (SEMC) facility. They were bred and housed in either the SPF or GF facility, with the temperature being maintained at approximately 23 °C under a 12-h light/12-h dark cycle. The light and dark periods started from ZT0 (7:30 am) and ZT12 (7:30 pm), respectively. The SPF mice were fed with a standard irradiated chow diet (Specialty Feeds, Perth, Australia), while GF mice were fed with an autoclaved standard chow diet (Laboratory Autoclavable Rodent Diet 5010, LabDiet, St. Louis, MO, USA). The mice were housed at a maximum of five mice per cage, with water given ad libitum. Only male mice were used in all experiments so as to eliminate confounding factors due to the estrous cycle. Mice were weighed before they were euthanized by carbon dioxide asphyxiation.

### 4.3. Antibiotic Treatments

Antibiotics were administered in drinking water to adult C57BL6J mice (*n* = 5 per antibiotic treatment group) for four weeks, as done previously in other studies [15,16,62,64]. Drinking water provided ad libitum was supplemented with antibiotics (Gold biotechnology, St. Louis, MO, USA) at 1 g/L for ampicillin (A-301-100), metronidazole (M-840-100), and neomycin sulfate (N-620-100), and at 0.5 g/L for vancomycin (V-200-25) [15]. In addition to the above treatment, another group of mice was treated with a concoction that consisted of all the above-mentioned antibiotics (AMNV), each at the same concentration as in individual antibiotic treatments [65]. The drinking water was changed twice a week for four weeks to selectively deplete the different spectra of the commensal gut flora [16,65,66]. The global characteristics of the antibiotics are as follows: ampicillin, is a semi-synthetic dermatophyte (e.g., fungus)-derived broad-spectrum antibiotic; metronidazole is a narrow spectrum nitroimidazole class antibiotic that targets anaerobic bacteria; neomycin is a broad-spectrum aminoglycoside that is particularly effective against gram-negative bacteria; and, vancomycin is a narrow spectrum glycopeptide antibiotic that eliminates gram-positive bacteria. Pharmacologically, ampicillin and metronidazole are moderately well absorbed from the gastrointestinal tract (GI) and are rapidly eliminated systemically within hours. On the contrary, the GI poorly absorbs vancomycin and neomycin [66].

Before euthanization, after four weeks of treatment with the respective antibiotics, faeces were collected by direct defaecation into sterile 2 mL microtubes at ZT4. Faecal samples were stored at −80 °C until subsequent analysis. Blood samples and livers were collected from the euthanized mice. On a given day, a total of six mice, one per treatment group, were harvested after four weeks of antibiotic treatment, starting from the first mouse harvest at ZT4 and the sixth mouse of the group latest at ZT6. For RNA and protein sample preparation, the tissues were frozen immediately after collection in liquid nitrogen until further processing.

### 4.4. Faecal DNA Analysis

Bacterial genomic DNA was extracted from 0.4–0.5 g of faecal samples using the FastDNA™ SPIN kit for faecal sample according to the manufacturer’s protocol (MP Biomedical, Aurora, CO, USA). Extracted DNA was resuspended in DNase/Pyrogen free water and the concentration was determined. DNA amplification and sequencing were performed by Chunlab Inc (Chunlab Inc, Seoul, Korea) [67]. DNA was first amplified using barcoded primers that were specific to the V1 and V3 regions of the bacterial 16S rRNA gene. Sequences were then sorted by their respective unique barcodes and the low-quality reads were removed. Bacterial 16S rRNA sequencing on 1 μg of PCR amplified product for each samples was done using the 454 GS FLX Titanium Sequencing Systems (Roche, Branford, CT, USA). The sequence reads were identified using the EzBioCloud Genome Database (Chunlab Inc, Seoul, Korea) [68]. Faecal composition, alpha diversity (species richness, diversity index, and coverage of data), and beta diversity (UPGMA clustering tree and PCoA) were analysed using the bioinformatics cloud platform, BIOiPLUG, as provided by Chunlab Inc (Chunlab Inc, Seoul, Korea).

### 4.5. Quantitative Reverse Transcriptase Polymerase Chain Reaction (RT-qPCR)

RNA was isolated from the liver using Trizol reagent and the PureLink® RNA Mini Kit (Life Technologies, Carlsbad, CA, USA), according to the manufacturer’s protocols. Purified RNA was spectrophotometrically quantified and RNA quality was assessed by the absorbance ratio at 260/280nm and 260/230nm using a Nanodrop Spectrophotometer (Thermo Fisher Scientific, Waltham, MA, USA). cDNA was synthesized from 1 μg of RNA by using the iScript reverse transcription (RT) supermix for RT-qPCR (Biorad, Hercules, CA, USA). The cDNA samples were then diluted for real-time polymerase chain reaction (PCR) using KAPA SYBR FAST qPCR Kits (KAPA Biosystems, Wilmington, MA, USA). Primers (Table S1) were ordered from Integrated DNA Technologies (IDT, Singapore, Singapore). Relative RNA levels were quantified using the ABI StepOnePlus RT-PCR System (Thermo Fisher Scientific, Waltham, MA, USA) in accordance with the KAPA Biosystem cycling condition. All of the amplified products were quantified based on the comparative delta-Ct method and then normalized to Glyceraldehyde-3-phosphate dehydrogenase (GAPDH) mRNA levels.

### 4.6. Statistical Analysis

The two-tailed Mann–Whitney test was performed on gene expression analysis using GraphPad Prism 7 (version 7.0b) software (GraphPad Software, San Diego, CA, USA). For the Mann–Whitney test, *p* value < 0.05 is considered to be statistically significant. Kruskal–Wallis followed by uncorrected Dunn’s multiple comparison was performed on alpha diversity data, *p* values < 0.05 is considered to be statistically significant. The correlation between faecal bacteria DNA load and cecum weight was calculated using a two-tailed Spearman correlation and *p* value < 0.05 is considered to be statistically significant.

### 4.7. D-DIGE

2D-DIGE was performed by Applied Biomics, Inc (Hayward, CA, USA). For every 10 mg of tissues, 200 μL of 2-D cell lysis buffer (30 mM Tris-hydrochloride (Tris-HCl), pH 8.8, containing 7 M urea, 2 M thiourea, and 4% 3-[(3-Cholamidopropyl)dimethylammonio]-1-propanesulfonate hydrate (CHAPS)) was added. Protein concentrations were measured using the Bio-Rad protein assay method. The internal standard was prepared by mixing equal amounts of protein from each sample and the protein spots from the labeled samples are compared to the internal standard for standardized abundances. CyDye labeling was performed for each sample by mixing 30 μg of protein with 0.5 μL of diluted CyDye and then kept in the dark on ice for 30 min. Samples from each gel were labeled with Cy2, Cy3, and Cy5, respectively. The labeling reaction was stopped by adding 1.0 μL of 10 mM lysine to each sample, and the samples were incubated in the dark on ice for an additional 15 min. The labeled samples were then mixed together. The 2X 2-D Sample buffer (8 M urea, 4% CHAPS, 2% pharmalytes and trace amount of bromophenol blue), 100 μL destreak solution, and rehydration buffer (7 M urea, 2 M thiourea, 4% CHAPS, 1% pharmalytes, and trace amount of bromophenol blue) were added to the labeling mix to make the total volume of 250 μL for the 13 cm immobilized pH gradient (IPG) strip. The labeled samples were mixed well and then spun before loading into the strip holder. After loading of the labeled samples, isoelectric focusing (IEF, pH 3–10, Non-Linear) was performed following the protocol that was provided by GE Healthcare (Pittsburgh, PA, USA). Upon finishing IEF, the IPG strips were incubated in the freshly made equilibration buffer-1 (50 mM Tris-HCl, pH 8.8, containing 6 M urea, 30% glycerol, 2% Sodium Dodecyl Sulfate (SDS), and trace amount of bromophenol blue) for 15 minutes with gentle shaking. The strips were subsequently rinsed in the freshly made equilibration buffer-2 (50 mM Tris-HCl, pH 8.8, containing 6 M urea, 30% glycerol, 2% SDS, and trace amount of bromophenol blue) for 10 min with gentle shaking. Next, the IPG strips were rinsed in the SDS-gel running buffer before being transferred into 10.5% SDS-gels. The SDS-gels were run at 15 °C until the dye front was running out of the gels. Gel images were scanned immediately following the SDS-PAGE using Typhoon TRIO (Applied Biomics, Inc, Hayward, CA, USA).. The scanned images were then analyzed by Image Quant software (Applied Biomics, Inc, Hayward, CA, USA ), followed by cross-gel analysis using the DeCyder software (Applied Biomics, Inc, Hayward, CA, USA). The fold change of the protein expression levels was obtained from DeCyder analysis.

### 4.8. Protein Identification by Mass Spectrometry

Applied Biomics, Inc. (Hayward, CA, USA) performed protein identification. The spots of interest were picked up using the Ettan Spot Picker (Amersham BioSciences, Buckinghamshire, UK) based on the in-gel analysis and spot picking design by DeCyder software (Applied Biomics, Inc Hayward, CA, USA). The gel spots were washed a few times, followed by in-gel digestion with modified porcine trypsin protease (Trypsin Gold, Promega, Madison, WI, USA). Digested tryptic peptides were desalted using Zip-tip C18 (Merck Millipore, Billerica, MA, USA). Peptides were eluted from the Zip-tip with 0.5 µL of matrix solution (α-cyano-4-hydroxycinnamic acid prepared in a concentration of 5 mg/mL in 50% acetonitrile, 0.1% trifluoroacetic acid, and 25 mM ammonium bicarbonate) and spotted on the AB SCIEX MALDI plate (Optic-TOF^TM^ 384 Well Insert) (AB SCIEX, Framingham, MA, USA). Matrix-Assisted Laser Desorption Ionization-Time of Flight Mass Spectrometry (MALDI-TOF MS) and TOF/TOF tandem MS were performed on an AB SCIEX TOF/TOF™ 5800 System (AB SCIEX, Framingham, MA, USA). MALDI-TOF mass spectra were acquired in the reflectron positive ion mode, averaging 4000 laser shots per spectrum. TOF/TOF tandem MS fragmentation spectra were acquired for each sample, averaging 4000 laser shots per fragmentation spectrum on each of the 10 most abundant ions that were present in each sample (excluding trypsin autolytic peptides and other known background ions). Both the resulting peptide mass and the associated fragmentation spectra were submitted to a GPS Explorer workstation equipped with the MASCOT search engine (Matrix Science, London, UK; http://www.matrixscience.com) to search the database of National Center for Biotechnology Information non-redundant (NCBInr, http://www.ncbi.nlm.nih.gov/RefSeq/) or Swiss-Prot database (https://www.uniprot.org/#). The searches were performed without constraining protein molecular weight or isoelectric point, with variable carbamidomethylation of cysteine and oxidation of methionine residues, and with one missed cleavage also being allowed in the search parameters. Candidates with either protein score C.I.% or Ion C.I.% greater than 95 were considered to be significant.

### 4.9. H-NMR Based Metabolomics

Liver polar extracts were prepared for NMR analysis according to the procedures that were described previously [69]. All 1H NMR spectra were obtained on a Bruker DRX-600-Avance NMR spectrometer (Bruker, Wissembourg, France) on the AXIOM metabolomics platform (MetaToul, Toulouse, France), operating at 600.13 MHz for 1H resonance frequency using an inverse detection 5-mm 1H-13C-15N cryoprobe that was attached to a cryoplatform (the preamplifier cooling unit). The 1H NMR spectra were acquired at 300K using a standard one-dimensional noesypr1D pulse sequence with water presaturation and a total spin-echo delay (2nτ) of 100 ms. A total of 128 transients were collected into 64,000 data points using a spectral width of 12 ppm, a relaxation delay of 2.5 s, and an acquisition time of 2.28 s. 1H-1H homonuclear correlation spectroscopy (COSY), 1H-1H Total Correlation Spectroscopy (TOCSY), and 1H-13C heteronuclear single quantum correlation experiment (HSQC) were obtained on one representative sample for metabolite identification.

Data were analyzed by applying an exponential window function with a 0.3-Hz line broadening prior to Fourier transformation. The resultant spectra were phased, baseline corrected, and then calibrated to Trimethylsilylpropanoic acid (TSP) (δ 0.00) manually using Mnova NMR v9.0(Mestrelab Research S.L., Santiago de Compostela, Spain). The spectra were subsequently imported into MatLab (R2014a, MathsWorks, Inc., Natick, MA, USA). All data were analyzed using full-resolution spectra. The region containing the water resonance (δ 4.6–5.2 ppm) was removed and the spectra were normalized to the probabilistic quotient [70] and then aligned using a previously published function [71]. The unsupervised PCA was used to see the distribution of the antibiotic-treated and non-treated groups, and the outliers were eliminated (one sample from SPF (Ampicillin) and one sample from GF (non-treated)). Data were mean-centered and scaled using the unit variance scaling prior to analysis using O-PLS-DA. 1H NMR data were used as independent variables (X matrix) and then regressed against a dummy matrix (Y matrix), indicating the class of samples (control or antibiotics). O-PLS-derived model was evaluated for goodness of prediction (Q2Y value) using n-fold cross-validation, where n depends on the sample size. To identify the metabolites that were responsible for discrimination between the groups, the O-PLS-DA correlation coefficients (r2) were calculated for each variable and then back-scaled into a spectral domain, so that the shape of NMR spectra and the sign of the coefficients were preserved [72]. The weights of the variables were color-coded, according to the square of the O-PLS-DA correlation coefficients. Correlation coefficients that were extracted from significant models were filtered so that only significant correlations above the threshold defined by Pearson’s critical correlation coefficient (*p* < 0.05; |r| > 0.87; for *n* = 5 per group) were considered to be significant. For illustration purpose, the area under the curve of several signals of interest was integrated and statistical significance was tested using the *t*-test.

## Supplementary Materials

Supplementary materials can be found at http://www.mdpi.com/1422-0067/20/4/812/s1.

## Abbreviations

2-D DIGE	2-Dimensional Fluorescence Difference Gel Electrophoresis
ACE	Abundance-based Coverage Estimator
AHR	Aryl Hydrocarbon Receptor
AMPK	AMP-activated Protein Kinase
BMAL1	Brain and Muscle Arnt-like Protein-1
CAR	Constitutive Androstane Receptor
CHAPS	3-[(3-Cholamidopropyl)Dimethylammonio]-1-Propanesulfonate Hydrate
CHREBP	Carbohydrate-Responsive Element-Binding Protein
CLOCK	Circadian Locomotor Output Cycles Kaput
CRY	Cryptochrome
COSY	homonuclear Correlation Spectroscopy
CYPs	Cytochrome P450s
DBP	Albumin Site D-Binding Protein
DEC2	Differentiated Embryonic Chondrocyte 2
E4BP4	Nuclear factor, Interleukin 3 Regulated
ECHS	Enoyl-CoA Hydratase
FABP5	Fatty Acid Binding Protein 5
FASN	Fatty Acid Synthase
FATS	Fragile-site Associated Tumour Suppressor
FPPS	Farnesyl Pyrophosphate Synthase
FXR	Farnesoid X Receptor
GAPDH	Glyceraldehyde-3-Phosphate Dehydrogenase
GF	Germ-Free
GR	Glucocorticoid Receptor
HLF	Hepatic Leukemia Factor
HSQC	Heteronuclear Single Quantum Correlation Spectroscopy
IACUC	Institutional Animal Care and Use Committee
IEF	Isoelectric Focusing
IPG	Immobilized pH Gradient
LPK	Liver Pyruvate Kinase
LXRα	Liver X Receptor Alpha
MALDI-TOF MS	Matrix-Assisted Laser Desorption Ionization-Time of Flight Mass Spectrometry
MTP	Microbiome Taxonomic Profiling
MUPs	Major Urinary Proteins
NMR	Nuclear Magnetic Resonance
O-PLS-DA	Orthogonal Projections to Latent Structures Discriminant Analysis
OTUs	Operational Taxonomic Units
PCA	Principal Component Analysis
PCoA	Principal Coordinate Analysis
PDK4	Pyruvate Dehydrogenase Kinase 4
PER	Period
PEPCK	Phosphoenolpyruvate Carboxykinase
PFN1	Profilin-1
PGC-1α	PPARγ-Coactivator-1 Alpha
PPARs	Peroxisome Proliferator-Activated Receptors
PXR	Pregnane X Receptor (NR1I2)
q-PCR	Quantitative Polymerase Chain Reaction
RT-qPCR	Quantitative reverse transcriptase polymerase chain reaction
RORs	Retinoid-related Orphan Receptors (NR1F)
REV-ERB	Rev-Erb receptors (NR1D)
SDS	Sodium Dodecyl Sulfate
SDS-PAGE	Sodium Dodecyl Sulfate Polyacrylamide Gel Electrophoresis
SEMC	Singhealth Experimental Medicine Centre
SHP	Small heterodimer partner
SIRT1	Sirtuin 1
SLC	Solute Carrier Transporter
SPF	Specific-pathogen Free
TEF	Thyrotroph embryonic factor
Tris-HCl	Tris-hydrochloride
TOCSY	Total Correlation Spectroscopy
TSP	Trimethylsilylpropanoic acid
UPGMA	Unweighted Pair Group Method with Arithmetic Mean
ZT	Zeitgeber Time
