# Supplementary material for: Depletion of Gram-Positive Bacteria Impacts Hepatic Biological Functions During the Light Phase
Source: Int J Mol Sci. 2019 Feb 14;20(4):812. doi: 10.3390/ijms20040812 (PMC6412208; doi:10.3390/ijms20040812)
Supplement: Supplementary file 1 [file ijms-20-00812-s001.pdf]

Primer	Primer Sequence (Forward 5'-3')	Primer Sequence (Reverse 5'-3')	NCBI Reference Sequence
Ahr	GCCCTTCCCGCAAGATGTTAT	TCAGCAGGGGTGGACTTTAAT	NM_013464
Ampk	TCTGAGGGGCACCAAGAAAC	GTGGGTGTTGACGGAGAAGAG	NM_001310480
Bmal1	ACAGTCAGATTGAAAAGAGG CG	GCCATCCTTAGCACGGTGAG	NM_001357070
Car	GCTGCAAGGGCTTCTTCAGA	CCTTCCAGCAAACGGACAGA	NM_001243063
Chrebp-β	TCTGCAGATCGCGTGGAG	CTTGTCCCGGCATAGCAAC	JQ437838
Clock	ACCGTAGCAGGTTTATGGGAA TG	TGGTGTCCACACAATAGGCAAGA	NM_001305222
Cry1	CAGACTCTCGTCAGCAAGATG	CAAACGTGTAAGTGCCTCAGT	NM_007771
Cry2	GCGTCTGTTTGTAGTCCGGG	TCCCAAAGGGTTCAGAGTCATA	NM_009963
Cyp1a1	CAATGAGTTTGGGGAGGTTAC TG	CCCTTCTCAAATGTCCTGTAGTG	NM_001136059
Cyp1a2	AGTACATCTCCTTAGCCCCAG	GGGTCCGGGTGGATTCTTC	NM_009993
Cyp2b10	TTTCTGCCCTTCTCAACAGGA A	ATGGACGTGAAGAAAAGGAACA AC	NM_009999
Cyp3a11	TCACACACACAGTTGTAGGCA GAA	GTTTACGAGTCCCATATCGGTAG AG	NM_007818
Cyp4a14	TCTCTGGCTTTTCTGTACTTTG CTT	CAGAAAGATGAGATGACAGGAC ACA	NM_007822
Cyp7a1	AGCAACTAAACAACCTGCCA GTACTA	GTCCGGATATTCAAGGATGCA	NM_007824
Cyp27a1	CCTTTGGGACTCGCACCA	GCCCTCCTGTCTCATCACTTG	NM_024264
Dbp	AAGAAGGCAAGGAAAGTCCA	TGTACCTCCGGCTCCAGTA	NM_016974
Dec2	GCGAGACGATACCAAGGATA C	TCAGATGTTCGGGCAGTAAA	NM_001271768
E4bp4	ACGGACCAGGGAGCAGAAC	GGACTTCAGCCTCTCATCCATC	NM_017373
Fabp5	TGAAAGAGCTAGGAGTAGGA CTG	CTCTCGGTTTTGACCGTGATG	NM_001272097
Fasn	AGTCAGCTATGAAGCAATTGT GGA	CACCCAGACGCCAGTGTTC	NM_007988
Fpps	GGAGGTCCTAGAGTACAATGC C	AAGCCTGGAGCAGTTCTACAC	NM_001253751

Fxr	TCCGGACATTCAACCATCAC	TCACTGCACATCCCAGATCTC	NM_001163700
Gapdh	TGGCCTTCCGTGTTCCTAC	GAGTTGCTGTTGAAGTCGCA	NM_001289726
Gr	CCGGGTCCCCAGGTAAAGA	TGTCCGGTAAAATAAGAGGCTTG	NM_008173
Hlf	CATCCTGAAGACGCATTTA	ATAAGGTGGGTCCCAAG	NM_172563
Lpk	TCGACTCAGAGCCTGTGGC	AGTCGTGCAATGTTCATCCCT	NM_013631
Lxrα	GGAGTGTCGACTTCGCAAATG	TCAAGCGGATCTGTTCTTCTGAC	NM_013839
Mup2	ATTAATGGGGAATGGCATACT A	GGATTCCATGCTCCTCACAT	NM_008647
Mup11	ATGAAGATGCTGTTGCTG	TCATTCTCGGGCCTTGAG	NM_001164526
Pdk4	ACCCTACGGATCCTAACCACC	TCACAGGCATTTTCTGAACCAAA G	NM_013743.2
Pepck	AACTGTTGGCTGGCTCTC	GAACCTGGCGTTGAATGC	NM_011044
Per1	GAATTGGAGCATATCACATCC GA	CCCGAAACACATCCCGTTTG	NM_001159367
Per2	AAAGCTGACGCACACAAAGA A	ACTCCTCATTAGCCTTCACCT	NM_011066
Pgc-1α	AAACTTGCTAGCGGTCCTCA	TGGCTGGTGCCAGTAAGAG	NM_008904
Pparα	TACTGCCGTTTTCACAAGTGC	AGGTCGTGTTCACAGGTAAGA	NM_011144
Pparβ	CGGCAGCCTCAACATGG	AGATCCGATCGCACTTCTCATAC	NM_011145
Pparγ	TGTGGGGATAAAGCATCAGG C	CCGGCAGTTAAGATCACACCTAT	NM_011146
Pxr	AGAGATCATCCCTCTTCTGCC AC	GATCTGGTCCTCAATAGGCAGGT	NM_010936
Rev-erbα	CTTCCGTGACCTTTCTCAGC	CAGCTCCTCCTCGGTAAGTG	NM_145434
Rev-erbβ	CGCCATGGAGCTGAACG	GACAAGAGGCAGGGCTGGA	NM_011584
Rorα	ACCGTGTCCATGGCAGAAC	TTTCCAGGTGGGATTTGGAT	NM_001289916
Rorβ	GGCAGACCCACACCTACGA	CAGAGCCTCCCTGGACTTG	NM_001289921
Rorγ	TCTACACGGCCCTGGTTCT	ATGTTCCACTCTCCTCTTCTCTTG	NM_001293734

Shp	CTCTGCAGGTCGTCCGACTAT TCTG	CCTCGAAGGTCACAGCATCCTG	NM_011850
Sirt1	CGGCTACCGAGGTCCATATAC	ACAATCTGCCACAGCGTCAT	NM_001159589
Slc37a4	GGCTACGGCTACTATCGCAC	GGAGAAGGTTTTGCGGTTGAAA	NM_001293630
Tef	GCCGAGCTTCGCAAGGA	ACAGGTTACAAGGGCCCGTACT	NM_153484

Supplementary Table 1. List of forward and reverse primers used for qPCR analysis of gene expression in mouse liver, with NCBI reference sequence indicated.

Assigned ID	Original No.	Protein Expression Ratio					
		V / C	Plus / C	M / C	Plus / V	M / V	M / Plus
1	40	-1.0	-1.3	1.3	-1.2	1.4	1.7
2	58	1.1	1.1	-1.4	-1.0	-1.6	-1.6
3	121	1.1	-1.4	1.3	-1.5	1.2	1.8
4	165	1.2	1.4	-1.2	1.2	-1.4	-1.6
5	150	1.2	1.4	-1.2	1.2	-1.3	-1.6
6	159	1.1	1.4	-1.2	1.2	-1.3	-1.6
7	135	1.1	1.3	-1.1	1.3	-1.2	-1.5
8	154	1.1	2.1	-1.0	1.8	-1.2	-2.2
9	161	1.4	2.3	1.2	1.6	-1.2	-1.9
10	131	1.0	1.4	-1.3	1.4	-1.3	-1.8
11	323	-1.0	-1.4	-1.6	-1.3	-1.6	-1.2
12	325	1.0	-1.4	-1.7	-1.4	-1.7	-1.2
13	326	1.1	-1.5	-1.7	-1.7	-1.9	-1.1
14	348	1.2	1.8	1.2	1.5	1.0	-1.5
15	443	1.1	1.4	-1.1	1.3	-1.2	-1.5
16	489	-1.3	-1.1	1.2	1.2	1.6	1.3
17	497	-1.3	-1.2	-1.9	1.1	-1.5	-1.6
18	583	1.2	-1.2	1.4	-1.4	1.2	1.7
19	596	-1.6	-1.7	-1.3	-1.0	1.2	1.3
20	665	-1.5	-1.1	-1.1	1.4	1.4	1.0
21	712	1.1	-1.1	-1.5	-1.2	-1.7	-1.4
22	717	1.2	-1.4	-2.3	-1.6	-2.6	-1.6
23	729	1.2	-1.5	-1.9	-1.8	-2.3	-1.3
24	721	1.4	-1.1	-1.5	-1.5	-2.0	-1.4
25	797	-1.1	1.3	-1.2	1.5	-1.0	-1.5
26	756	-1.1	-1.2	1.3	-1.1	1.5	1.6
27	757	-1.0	-1.2	1.3	-1.1	1.4	1.5
28	769	1.1	-1.2	-1.6	-1.2	-1.6	-1.4
29	814	-1.1	1.2	-1.4	1.4	-1.2	-1.7
30	818	-1.0	2.1	1.1	2.1	1.2	-1.8
31	827	1.1	1.8	-1.3	1.6	-1.5	-2.4
32	825	-1.2	-1.5	1.2	-1.3	1.4	1.8
33	857	1.6	1.1	1.5	-1.4	-1.0	1.4
34	987	1.2	1.0	-1.3	-1.1	-1.5	-1.4

35	1013	1.2	1.9	-1.2	1.6	-1.4	-2.2
36	1076	1.6	1.4	1.1	-1.1	-1.5	-1.3
37	1007	1.1	-1.6	-1.5	-1.7	-1.6	1.1
38	1034	1.3	-1.0	-1.2	-1.3	-1.5	-1.2
39	1036	-1.0	-1.5	-1.5	-1.5	-1.5	-1.0
40	1035	1.1	-1.3	1.2	-1.4	1.2	1.6
41	1048	1.9	2.3	1.4	1.2	-1.4	-1.7
42	1056	1.7	1.6	1.4	-1.1	-1.2	-1.1
43	1064	1.6	1.4	1.3	-1.2	-1.3	-1.1
44	1094	-1.1	-1.2	1.4	-1.1	1.5	1.7
45	1195	-1.5	-1.2	-1.1	1.3	1.3	1.1
46	1145	-1.2	-1.2	1.4	1.0	1.7	1.7
47	1127	1.5	1.0	1.3	-1.5	-1.2	1.2
48	1180	-1.5	-1.6	-1.8	-1.1	-1.2	-1.1
49	1217	-1.1	1.3	-1.3	1.3	-1.2	-1.6
50	1200	-1.8	1.8	1.4	3.2	2.6	-1.2
51	1273	-1.2	1.4	1.3	1.7	1.6	-1.1
52	1348	-1.1	1.5	-1.1	1.6	-1.1	-1.7
53	1473	-1.4	-1.1	1.1	1.4	1.6	1.2
54	1471	-1.8	-1.1	-1.1	1.6	1.6	1.0
55	1472	1.0	-1.1	1.4	-1.2	1.3	1.6
56	1476	1.1	-1.1	1.9	-1.2	1.8	2.1
57	1520	-1.0	1.1	1.7	1.1	1.7	1.5
58	1549	-1.1	1.2	1.3	1.4	1.5	1.1
59	1519	1.5	1.5	1.1	-1.0	-1.4	-1.3
60	1577	1.5	-1.2	1.4	-1.7	-1.1	1.6
61	1602	-1.4	-1.1	-1.7	1.3	-1.2	-1.6
62	1614	-1.1	-1.7	1.0	-1.5	1.2	1.8
63	1580	-1.3	1.1	1.2	1.5	1.6	1.1
64	1561	-1.2	-1.3	-1.8	-1.1	-1.5	-1.4
65	1609	1.9	1.2	1.7	-1.6	-1.1	1.5
66	1732	-1.6	-4.5	-2.3	-2.8	-1.4	1.9
67	1728	-1.6	-5.0	-2.7	-3.2	-1.7	1.9
68	1758	-1.8	-2.5	-1.4	-1.4	1.3	1.8
69	1750	-2.7	-3.0	-1.5	-1.1	1.8	2.1
70	1778	6.5	-1.2	2.9	-8.0	-2.3	3.5
71	1793	-2.0	-3.1	-1.7	-1.6	1.2	1.8
72	1788	-1.9	-2.7	-1.8	-1.4	1.1	1.5
73	1821	-1.8	-1.3	-2.5	1.3	-1.4	-1.8
74	1780	1.2	1.1	-1.3	-1.1	-1.6	-1.5
75	1869	-1.6	1.3	-1.3	2.1	1.2	-1.7
76	1890	-1.0	-1.3	2.2	-1.3	2.2	2.8
77	1916	-1.1	1.4	1.9	1.5	2.0	1.3
78	1961	-1.7	-1.5	-1.8	1.1	-1.1	-1.2
79	1945	1.8	-1.1	1.8	-1.9	1.0	1.9
80	1957	1.3	1.1	-2.0	-1.1	-2.5	-2.3
81	2007	-1.0	-1.5	1.2	-1.5	1.3	1.9
82	2046	2.8	-1.1	1.8	-3.1	-1.6	2.0
83	2053	6.2	1.6	4.5	-4.0	-1.4	2.8
84	2045	2.2	1.2	1.8	-1.9	-1.2	1.6
85	2048	6.7	-1.1	4.9	-7.1	-1.4	5.2
86	2036	-1.7	-1.7	-3.4	1.1	-2.0	-2.1
87	2034	1.1	-1.4	1.3	-1.4	1.2	1.8
88	307	-1.3	-1.4	-1.1	-1.1	1.2	1.3
89	312	1.4	1.4	1.3	1.0	-1.0	-1.1

90	308	1.1	-1.1	1.0	-1.2	-1.1	1.1
91	313	1.4	1.5	1.2	1.1	-1.2	-1.3
92	364	1.2	1.7	1.3	1.5	1.1	-1.4
93	357	1.2	1.5	1.2	1.3	1.0	-1.3
94	1506	-1.0	1.2	5.1	1.3	5.2	4.1
95	1741	-1.8	-3.1	-2.5	-1.7	-1.4	1.3
96	1805	-2.2	-2.2	-3.0	1.0	-1.4	-1.4

Supplementary Table 2. List of all protein spots with ID assigned identified by mass spectrometry and selected from 2D-DIGE (see Fig. 4). Respective fold change among the different treatment groups obtained from DeCyder analysis is shown.

Number	Metabolite	Metabolic pathway	δ ^{1}H (ppm)	Multiplicity
1	Bile acids (mixed)	Bile component	0.6-0.75	s
			0.92	s
2	Bile acids (tauroconjugated)	Bile component	0.72	s
			0.92	s
			3.08	t
3	Leucine	Amino acid	0.96	t
			1.72	m
			3.74	m
4	Valine	Amino acid metabolism	0.99	d
			1.05	d
			2.29	

			3.62	
5	Isoleucine	Amino acid	0.94	t
			1.01	d
			1.26	m
			1.48	m
			2	m
			3.65	d
6	3-hydroxybutyrate	Ketone body	1.19	d
			2.32	dd
			2.4	dd
			4.14	m
7	Lactate	Organic acids - Energy metabolism	1.32	d
			4.11	q
8	Threonine	Amino acid metabolism	1.33	d
			3.61	
			4.23	
9	Alanine	Amino acid metabolism	1.49	d
			3.79	
10	Ornithine	Amino acid metabolism	1.72	m
			1.93	m

			3.03	t
			3.77	t
11	Acetate	Organic acids - Energy metabolism	1.93	s
12	L-glutamate	Amino acid metabolism	2.06	m
			2.35	m
			3.76	dd
13	L-glutamine	Amino acid metabolism	2.13	m
			2.45	m
			3.77	t
14	Glutathion (oxidized)	Glutathion metabolism	2.17	t
			2.53	m
			2.98	dd
			3.31	m
			3.76	m
			4.75	m
15	Glutathion (reduced)	Glutathion metabolism	2.17	m
			2.56	m
			2.95	m
			3.76	m

			4.56	dd
16	Succinate	Organic acids - Energy metabolism	2.41	s
17	L-aspartic acid	Amino acid metabolism	2.66	dd
			2.8	dd
			3.89	dd
18	Dimethylamine	Choline metabolism - Host-gut microbiota cometabolism	2.72	s
19	Unknown 1		2.84	t
			3.62	m
20	Dimethylglycine	Choline-betaine metabolism - One carbon metabolism	2.93	s
21	Creatine	Muscle energy metabolism	3.04	s
			3.94	s
			4.07	s
22	Choline	Choline-betaine metabolism - One carbon metabolism	3.2	s
			3.55	

23	Glycerophosphocholine	Choline-betaine metabolism - One carbon metabolism	3.23	s
24	O-phosphocholine	Choline-betaine metabolism - One carbon metabolism	3.21	s
			3.58	m
			4.13	m
25	Betaine	Amino acid metabolism	3.27	s
			3.88	s
26	Taurine	Amino acid metabolism	3.28	t
			3.44	t
27	Methanol		3.36	s
28	β-glucose	Carbohydrate metabolism	3.51	
			3.75	
			4.66	d
29	AMP		4.01	dd
			4.36	dd
			4.5	dd
			6.14	d
			8.27	s

			8.62	s
30	α-glucose	Carbohydrate metabolism	3.45	
			3.56	
			3.72	
			3.84	
			3.97	
			5.25	d
31	Glycine	Amino acid metabolism	3.56	s
32	UDP-glucose	Carbohydrate metabolism	5.6	dd
			5.95	d
			7.93	d
33	UDP-glucuronate	Carbohydrate metabolism	5.6	dd
			5.98	d
			7.96	d
34	Uridine		5.89	d
			5.9	d
			7.89	d
35	NADP+	Coenzyme	6.03	s
			6.1	d
			8.15	d

			8.42	s
			8.584	s
			8.82	s
			9.12	d
			9.3	s
36	NAD+	Coenzyme	6.04	d
			6.09	d
			8.18	s
			8.2	dd
			8.44	s
			8.84	d
			9.15	d
			9.34	s
37	Inosine	Purine metabolism	6.11	d
			8.23	s
			8.34	s
38	Fumarate	Energy metabolism	6.52	s
39	Tyrosine	Amino acid metabolism	6.87	d
			7.19	
40	Phenylalanine	Amino acid metabolism	3.12	dd
			3.26	dd
			7.33	m

			7.38	m
			7.43	m
41	Nicotinurate	Nicotinate - nicotinamide metabolism	3.99	s
			7.6	dd
			8.25	m
			8.72	dd
			8.94	s
42	Formate	Organic acids - One-carbon metabolism	8.45	s

Supplementary Table 3. Table showing ^{1}H chemical shift assignments of liver metabolites observed in the NMR spectra with the metabolic pathways implicated. Metabolites are identified with their respective parts per million (ppm) and multiplicity as indicated.

**Family**	**Amount present in Feces (%)**					
	**C**	**N**	**M**	**V**	**A**	**AMNV**
AB096215_f	-	-	-	-	0.01	-
AB186887_f	-	-	-	-	0.00	-
AB240310_f	-	-	-	-	0.01	-
AB240334_f	-	-	-	-	0.01	-
AB257647_f	-	-	-	-	0.01	-

AB374370_f	-	-	-	-	0.02	-
AB511016_f	-	-	-	-	0.17	-
AB672201_f	-	-	-	-	0.01	-
AB672277_f	-	-	-	-	0.01	-
AF269002_f	-	-	-	-	0.01	-
AF358012_f	-	-	-	-	0.02	-
AF407708_f	-	-	-	-	0.02	-
AF498716_f	-	-	-	-	0.45	-
AM275436_f	0.04	-	0.04	-	0.03	-
AM275436_o_uc	-	-	0.01	-	-	-
AM991231_f	-	-	-	-	0.01	-
ASND_f	-	-	-	-	0.01	-
AY234747_f	-	-	-	-	0.06	-
AY281358_f	-	-	-	0.02	0.01	0.01
AY289487_f	-	-	-	-	0.01	-
AY673403_f	-	-	-	-	0.05	-
AY673403_o_uc	-	-	-	-	0.01	-
AY945895_f	-	-	-	-	0.00	-
Acetobacteraceae	-	-	-	-	0.11	-
Acholeplasmataceae	-	-	-	0.44	-	-
Acidaminococcaceae	-	-	-	-	0.03	-
Acidiferrobacteraceae	-	-	-	-	0.02	-
Acidimicrobiaceae	-	-	-	-	0.02	-
Acidobacteriaceae	-	-	-	-	0.50	-

Actinomycetaceae	-	-	-	-	0.53	-
Actinospicaceae	-	-	-	-	0.01	-
Aerococcaceae	-	-	-	-	0.14	-
Aeromonadaceae	-	-	-	-	0.01	-
Afifella_f	-	-	-	-	0.01	-
Akkermansiaceae	0.82	2.14	1.59	3.76	0.12	-
Alcaligenaceae	-	-	-	-	0.01	-
Alsobacter_f	-	-	-	-	0.02	-
Anacardiaceae	-	-	-	-	0.00	-
Anaerolinaceae	0.01	-	-	-	0.01	-
Anaeromyxobacteraceae	-	-	-	-	0.02	-
Anaplasmataceae	-	-	-	-	0.01	-
Aneurinibacillus_f	-	-	-	-	0.01	-
Annonaceae	-	-	-	-	0.03	-
Araceae	-	-	-	-	0.00	-
Archangiaceae	-	-	-	-	0.02	-
Armatimonadaceae	-	-	-	-	0.04	-
Asparagaceae	-	-	-	-	0.04	-
Atherospermataceae	-	-	-	-	0.01	-
Aurantimonadaceae	-	-	-	-	0.02	-
Bacillaceae	-	-	-	-	5.49	-
Bacillales_uc	0.01	-	-	-	-	-
Bacteroidaceae	0.50	1.57	6.48	-	0.99	-
Bacteroidales_uc	0.06	0.08	0.02	0.01	-	-

Bdellovibrionaceae	-	-	-	-	0.00	-
Beggiatoaceae	-	-	-	-	0.01	-
Beijerinckiaceae	-	-	-	-	0.16	-
Bifidobacteriaceae	0.70	1.11	2.51	-	0.28	0.00
Bifidobacteriales_uc	-	-	0.01	-	-	-
Blastocatellaceae	-	-	-	-	0.01	-
Bogoriellaceae	-	-	-	-	0.01	-
Bosea_f	-	-	-	-	0.01	-
Bradyrhizobiaceae	-	-	-	-	0.47	-
Brassicaceae	-	-	-	-	0.08	-
Brevibacteriaceae	-	-	-	-	0.18	-
Brucellaceae	-	-	-	-	0.02	-
Burkholderiaceae	-	-	-	-	3.90	-
Burkholderiales_uc	0.00	-	0.01	-	-	-
Buxaceae	-	-	-	-	0.01	-
CP006913_f	-	-	-	-	0.02	-
CP009312_f	-	-	-	-	0.01	-
CP011215_f	-	-	-	-	0.06	-
CP011215_o_uc	-	-	-	-	0.01	-
CP011489_f	-	-	-	-	0.01	-
CP012157_f	-	-	-	-	0.04	-
CP015136_f	-	-	-	-	0.12	-
Caldicoprobacteraceae	-	-	-	-	0.01	-
Calycanthaceae	-	-	-	-	0.00	-

Campylobacteraceae	-	-	-	-	0.29	-
Cardiobacteriaceae	-	-	-	-	0.29	-
Carnobacteriaceae	-	0.02	-	-	0.08	-
Catenulisporaceae	-	-	-	-	0.02	-
Caulobacteraceae	-	-	-	-	0.38	-
Cellulomonadaceae	-	-	-	-	0.00	-
Cellvibrionales_uc	-	-	-	0.00	-	-
Chitinophagaceae	-	-	0.01	-	0.59	0.00
Chloranthaceae	-	-	-	-	0.01	-
Christensenellaceae	0.18	0.06	0.02	1.46	0.10	0.00
Chroococcidiopsis_f	-	-	-	-	0.00	-
Chthoniobacteraceae	-	-	-	-	0.01	-
Chthonomonadaceae	-	-	-	-	0.00	-
Clostridiaceae	0.01	-	0.01	-	0.31	0.00
Clostridiales_uc	0.05	0.03	-	0.04	-	-
Cohaesibacteraceae	-	-	-	0.00	-	-
Comamonadaceae	-	-	-	-	1.52	-
Conexibacteraceae	-	-	-	-	0.11	-
Cordycipitaceae	-	-	-	-	0.00	-
Coriobacteriaceae	1.09	0.02	0.21	-	0.13	-
Coriobacteriales_uc	0.01	-	-	-	-	-
Corynebacteriaceae	-	-	-	-	1.96	0.00
Coxiellaceae	-	-	-	-	0.03	-
Crassulaceae	-	-	-	-	0.06	-

Crocinitomicaceae	-	-	-	-	0.04	-
Cryptosporangiaceae	-	-	-	-	0.01	-
Cytophagaceae	-	-	-	-	0.03	-
DQ129389_f	-	-	-	-	0.01	-
DQ154856_f	-	-	-	-	0.00	-
DQ228400_f	-	-	-	-	0.01	-
DQ394955_f	-	-	-	-	0.03	-
DQ395705_f	-	-	-	-	0.02	-
DQ404819_f	-	-	-	-	0.04	-
DQ413083_f	-	-	-	-	0.01	-
DQ676361_f	-	-	-	-	0.03	-
DQ906813_f	-	-	-	-	0.05	-
Deferribacteraceae	-	-	-	-	0.02	-
Deinococcaceae	-	-	-	-	0.05	-
Dermabacteraceae	-	-	-	-	0.42	-
Desulfovibrionaceae	0.27	-	-	0.02	-	-
Devosia_f	-	-	-	-	0.11	-
Dietziaceae	-	-	-	-	0.10	-
EF445272_f	1.08	-	0.49	-	0.01	-
EF516242_f	-	-	-	-	0.01	-
EF516466_f	-	-	-	-	0.01	-
EF516615_f	-	-	-	-	0.01	-
EF516692_f	-	-	-	-	0.02	-
EU133950_f	-	-	-	-	0.01	-

EU133950_o_uc	-	-	-	-	0.01	-
EU234093_f	0.07	-	0.11	-	0.00	-
EU289437_f	-	-	-	-	0.01	-
EU335161_f	-	-	-	-	0.00	-
EU335336_f	-	-	-	-	0.01	-
EU445199_f	-	-	-	0.00	0.22	-
EU491430_f	-	-	-	-	0.01	-
EU644175_f	-	-	-	-	0.04	-
EU644175_o_uc	-	-	-	-	0.01	-
EU680443_f	-	-	-	-	0.01	-
EU786132_f	-	-	-	-	0.01	-
EU845084_f	0.18	0.03	4.87	-	0.26	-
EU861904_f	-	-	-	-	0.01	-
EU881211_f	-	-	-	-	0.02	-
Enterobacteriaceae	0.01	-	0.44	6.13	0.48	99.94
Enterobacteriales_uc	-	-	-	0.01	-	0.00
Enterococcaceae	-	-	-	-	1.87	0.00
Erysipelotrichaceae	9.50	2.89	50.70	0.00	3.21	-
Erysipelotrichales_uc	4.24	0.86	0.39	-	0.05	-
Erythrobacteraceae	-	-	-	-	0.05	-
Eubacteriaceae	0.02	0.01	-	-	-	-
Euphorbiaceae	-	-	-	-	0.01	-
Exiguobacteriaceae	-	-	-	-	0.01	-
FJ625377_f	-	-	-	-	0.00	-

FJ889281_f	-	-	-	-	0.08	-
FM253572_f	-	-	-	-	0.01	-
FR687426_f	-	-	-	-	0.01	-
FR749824_f	-	-	-	-	0.01	-
FR888536_f	0.53	0.25	-	-	-	-
Fabaceae	-	-	-	-	0.56	-
Fimbriimonadaceae	-	-	-	-	0.02	-
Flavobacteriaceae	-	-	-	-	1.93	-
Frankiaceae	-	-	-	-	0.09	-
Fusobacteriaceae	-	-	-	-	0.85	-
GQ263151_f	-	-	-	-	0.02	-
GQ472436_f	-	-	-	-	0.01	-
GU199451_f	-	-	-	-	0.06	-
GU444092_f	-	-	-	-	0.04	-
GU983350_f	-	-	-	-	0.02	-
Gaiellaceae	-	-	-	-	0.11	-
Gemella_f	-	-	-	-	0.05	-
Geminigeraceae	-	-	-	-	0.01	-
Gemmata_f	-	-	-	-	0.09	-
Gemmatimonadaceae	-	-	-	-	0.13	-
Geodermatophilaceae	-	-	-	-	0.01	-
HM748665_f	-	-	-	-	0.01	-
HM748667_o_uc	-	-	-	-	0.01	-
HQ178786_f	-	-	-	-	0.04	-

HQ645210_f	-	-	-	-	0.02	-
HQ674891_f	-	-	-	-	0.02	-
HQ681992_f	-	-	-	-	0.04	-
HQ910322_f	-	-	-	-	0.02	-
Haliangiaceae	-	-	-	-	0.09	-
Helicobacteraceae	0.02	-	-	-	-	-
Holophagaceae	-	-	-	-	0.05	-
Hydrogenispora_o_uc	0.00	-	-	-	-	-
Hydrogenophilaceae	-	-	-	-	0.01	-
Hyphomicrobiaceae	-	-	-	-	0.06	-
Hypocreaceae	-	-	-	-	0.00	-
Hypocreales_uc	-	-	-	-	0.03	-
Ilumatobacter_f	-	-	-	-	0.03	-
Intrasporangiaceae	-	-	-	-	0.18	-
JF319233_f	-	-	-	-	0.00	-
JF417809_f	-	-	-	-	2.73	-
JF421159_f	-	-	-	-	0.11	-
JF703539_f	-	-	-	-	0.00	-
JF737898_f	-	-	-	-	0.16	-
JF776882_f	-	-	-	-	0.01	-
JF922436_f	-	-	-	-	0.00	-
JQ650114_f	-	-	-	-	0.07	-
JX133647_f	-	-	-	-	0.02	-
Ktedonobacteraceae	-	-	-	-	0.04	-

LCGL_o_uc	-	-	-	-	0.01	-
Lachnospiraceae	26.41	22.74	0.81	-	2.16	0.01
Lactobacillaceae	9.98	0.24	4.54	20.64	2.12	0.00
Lactobacillales_uc	0.01	-	0.00	-	-	-
Lautropia_f	-	-	-	-	0.31	-
Legionellaceae	-	-	-	-	0.04	-
Lentisphaeraceae	-	-	-	0.00	-	-
Leptotrichiaceae	-	-	-	-	1.64	-
Leuconostocaceae	-	-	-	-	0.59	-
Luteolibacter_f	-	-	-	-	0.01	-
Methylobacteriaceae	-	-	-	-	0.16	-
Methylocystaceae	-	-	-	-	0.01	-
Methylophilaceae	-	-	-	-	0.08	-
Micavibrio_f	-	-	-	-	0.02	-
Microbacteriaceae	-	-	-	-	0.25	-
Micrococcaceae	-	-	-	-	0.32	-
Micromonosporaceae	-	-	-	-	0.12	-
Mogibacterium_f	0.20	0.02	0.07	-	0.04	-
Moraxellaceae	-	-	-	-	0.11	-
Mycobacteriaceae	-	-	-	-	0.06	-
Mycoplasmataceae	-	-	-	-	0.01	-
Nakamurellaceae	-	-	-	-	0.02	-
Natronincola_f	-	-	-	-	0.01	-
Neisseriaceae	-	-	-	-	1.35	-

Nitrospiraceae	-	-	-	-	0.06	-
Nocardiaceae	-	-	-	-	0.02	-
Nocardioidaceae	-	-	-	-	0.06	-
Nocardiopsaceae	-	-	-	-	0.01	-
OMAN_f	-	-	-	-	0.02	-
Oceanospirillaceae	-	-	-	0.00	-	-
Odoribacteraceae	0.06	0.01	-	-	-	-
Opitutaceae	-	0.11	-	-	0.01	-
Oxalobacteraceae	-	-	-	-	0.29	-
Paenibacillaceae	-	-	-	-	0.28	-
Parvibaculum_f	-	-	-	-	0.05	-
Pasteurellaceae	-	-	-	-	1.23	-
Pedosphaera_f	-	-	-	-	0.07	-
Pelagibacteraceae	-	-	-	-	0.28	-
Peptococcaceae	-	-	-	-	0.02	-
Peptoniphilaceae	-	-	-	-	0.03	-
Peptostreptococcaceae	-	-	-	-	0.18	-
Phreatobacter_f	-	-	-	-	0.01	-
Phycisphaeraceae	-	-	-	-	0.02	-
Phyllobacteriaceae	-	-	-	-	28.09	-
Pinaceae	-	-	-	-	0.07	-
Planctomycetaceae	-	-	-	-	0.05	-
Planktophila_f	-	-	-	-	0.05	-
Planococcaceae	-	-	-	-	0.77	0.00

Poaceae	-	-	-	-	0.52	-
Polyangiaceae	-	-	-	-	0.09	-
Porphyromonadaceae	0.94	8.14	2.24	65.86	1.29	-
Prevotellaceae	2.33	2.20	0.56	-	1.93	-
Prochlorococcaceae	-	-	0.00	-	-	-
Propionibacteriaceae	-	-	-	0.00	0.70	0.00
Pseudanabaenaceae	-	-	-	-	0.00	-
Pseudoalteromonadaceae	-	-	-	-	0.57	0.00
Pseudomonadaceae	-	-	-	-	0.16	0.00
Pseudonocardiaceae	-	-	-	-	0.14	-
Puniceicoccaceae	-	-	-	-	0.04	-
Pyrenomonadales_uc	-	-	-	-	0.01	-
Ralstonia_f	-	-	-	-	4.47	-
Rhizobiaceae	-	-	-	-	0.27	-
Rhizomicrobium_f	-	-	-	-	0.12	-
Rhodobacteraceae	-	-	-	-	0.17	0.00
Rhodospirillaceae	-	-	0.26	-	0.34	-
Rickettsiaceae	-	-	-	-	0.01	-
Rikenellaceae	1.78	2.92	0.21	-	0.04	-
Ruminococcaceae	13.67	2.78	1.49	-	1.01	-
S24-7_f	24.66	50.91	13.34	0.00	2.27	-
Saccharimonas_f	0.15	-	-	-	0.41	-
Sandaracinaceae	-	-	-	-	0.02	-
Sanguibacteraceae	-	-	-	-	0.04	-

Saururaceae	-	-	-	-	0.01	-
Sedimenticola_f	-	-	-	-	0.00	-
Selenomonadaceae	-	-	-	-	0.23	-
Shewanellaceae	-	-	-	-	0.00	-
Sinobacteraceae	-	-	-	-	0.02	-
Solanaceae	-	-	-	-	0.03	-
Solibacteraceae	-	-	-	-	0.12	-
Sphingobacteriaceae	-	-	-	-	0.12	-
Sphingomonadaceae	0.00	-	-	-	0.23	-
Spirochaetaceae	-	-	-	-	0.08	-
Sporichthyaceae	-	-	-	-	0.00	-
Staphylococcaceae	0.02	-	-	-	3.26	0.00
Steroidobacter_f	-	-	-	-	0.14	-
Sterolibacterium_f	-	-	-	-	0.02	-
Streptococcaceae	0.01	-	-	-	3.22	-
Streptomycetaceae	-	-	-	-	0.01	-
Sutterellaceae	0.33	0.78	8.53	1.58	0.41	-
Tepidisphaeraceae	-	-	-	-	0.06	-
Thermoactinomycetaceae	-	-	-	-	0.03	-
Thermoleophilaceae	-	-	-	-	0.02	-
Thermomonosporaceae	-	-	-	-	0.03	-
Thiobacillus_f	-	-	-	-	0.01	-
Tissierellaceae	-	-	-	-	0.04	-
Tofieldiaceae	-	-	-	-	0.00	-

Trimeniaceae	-	-	-	-	0.01	-
Veillonellaceae	-	-	-	-	0.12	-
Verrucomicrobiaceae	-	-	-	-	0.00	-
Verrucomicrobiales_uc	-	-	-	0.00	-	-
Vibrionaceae	-	-	-	-	0.17	0.00
Vibrionales_uc	-	-	-	-	-	0.00
Xanthomonadaceae	-	-	-	-	0.29	-
Yaniellaceae	-	-	-	-	0.01	-

Supplementary Table 4. Families of bacteria sequenced from feces of Control (C, white), Neomycin (N, green), Metronidazole (M, yellow), Vancomycin (V, blue), Ampicillin (A, red) and combination (AMNV, grey) and identified with their abundance represented by the percentage.

Supplementary Figure 1

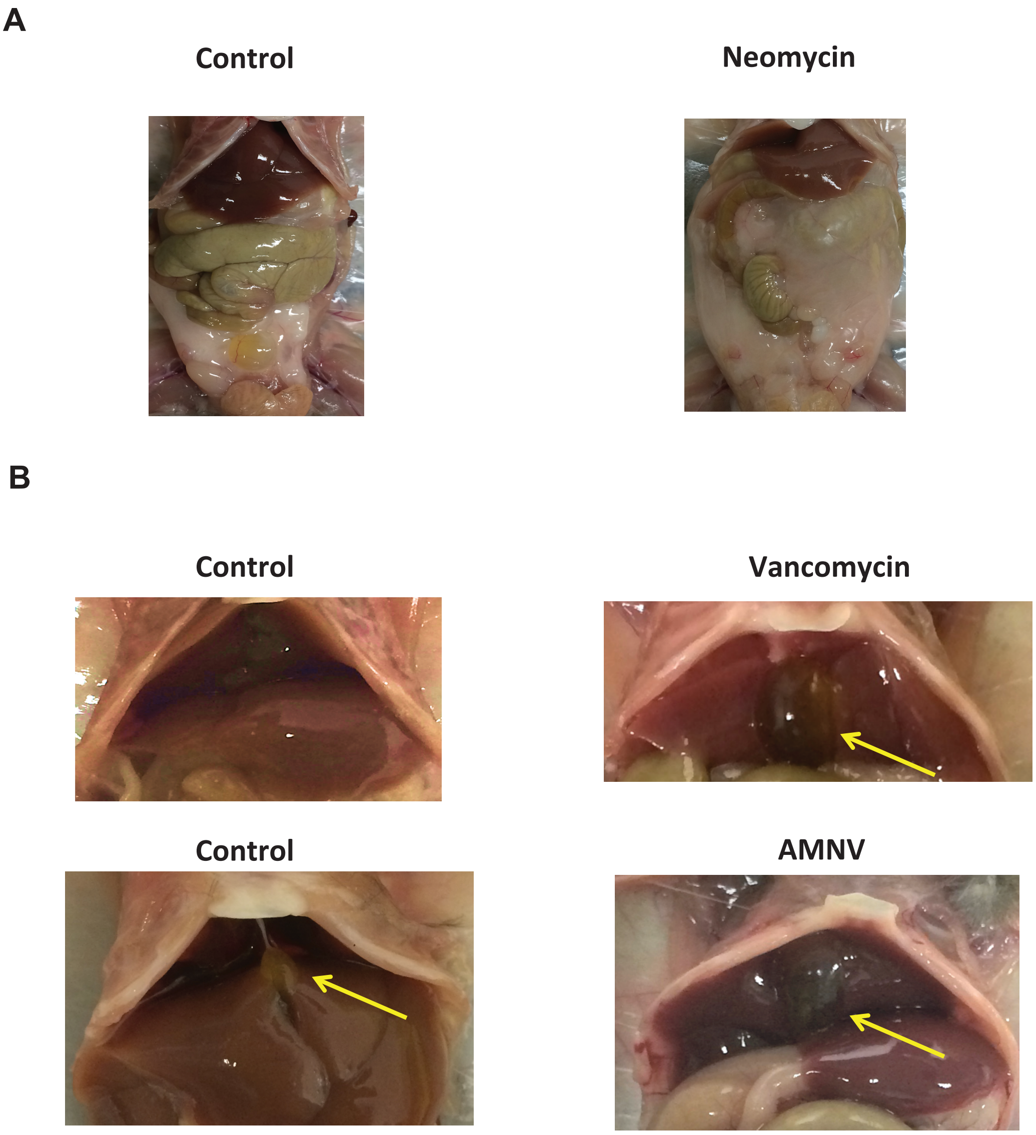

Supplementary Figure 1. Gross evaluation of mice that underwent 4 weeks of respective antibiotic treatments administered via drinking water ad libitum. (**A**) Images of control and neomycin treated mice showing accumulation of excess visceral fat upon one month of neomycin treatment. (**B**) Images of control, vancomycin and AMNV treated mice showing dilated gallbladder after vancomycin and AMNV treatment. Gallbladder in the control group was not visible (control upper image) and liver lobes had to be adjusted to view the size of the gallbladder (control lower image). Yellow arrow: dilated gallbladder.

Supplementary Figure 2

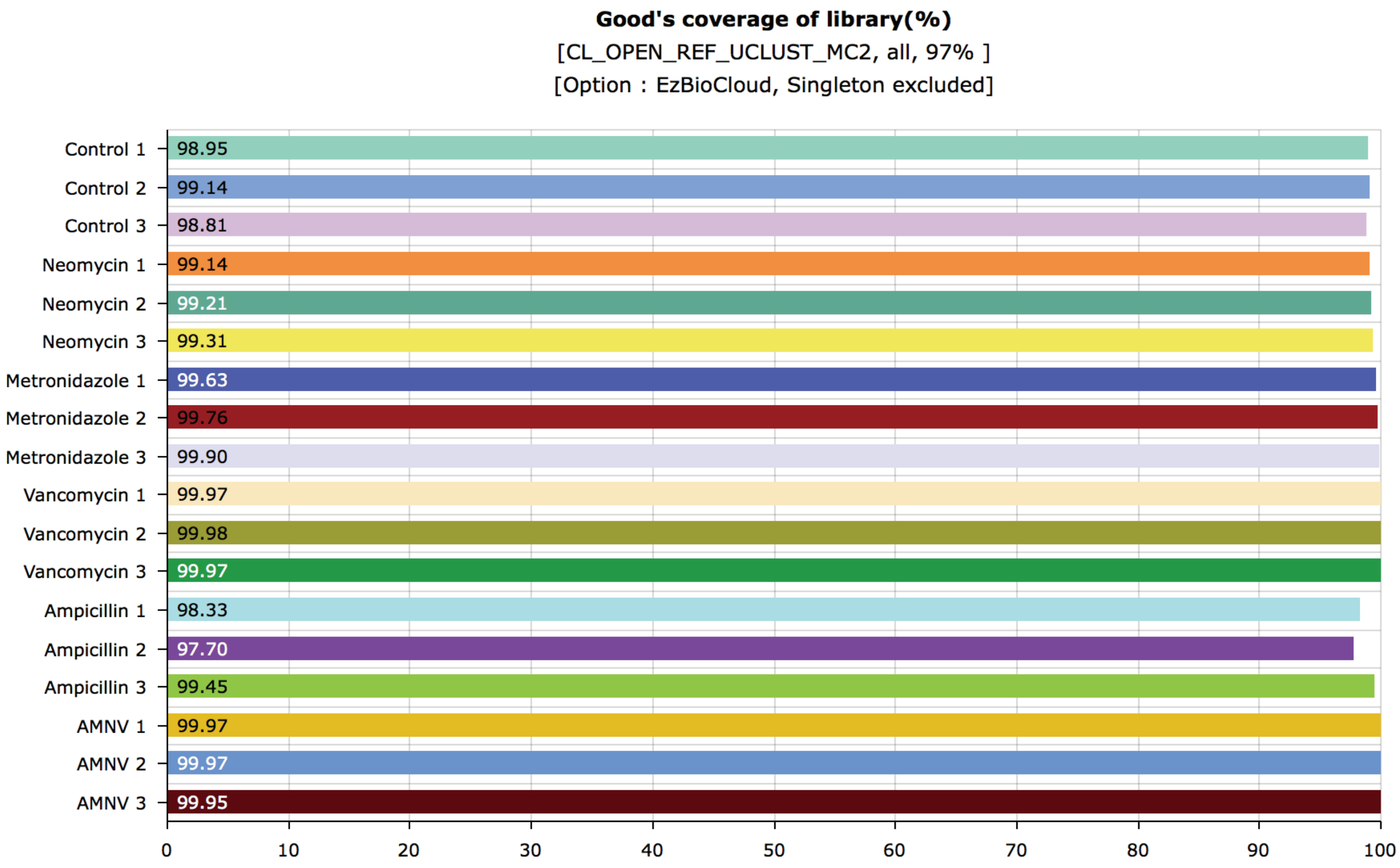

Supplementary Figure 2. Goods coverage of library. Estimates with a minimum of 97.7% for pyrosequencing of faecal samples indicated good library coverage. Singletons (sequence read that is present exactly once) were excluded from the analysis to eliminate possible taxonomic artifacts.
